# Pulmonary Delivery of Virosome-Bound Antigen Enhances Antigen-Specific CD4^+^ T Cell Proliferation Compared to Liposome-Bound or Soluble Antigen

**DOI:** 10.3389/fimmu.2017.00359

**Published:** 2017-04-07

**Authors:** Rebecca A. M. Blom, Mario Amacker, R. Maarten van Dijk, Christian Moser, Philip A. Stumbles, Fabian Blank, Christophe von Garnier

**Affiliations:** ^1^Department of Pulmonary Medicine, Inselspital, Bern University Hospital, University of Bern, Bern, Switzerland; ^2^Department of Clinical Research, University of Bern, Bern, Switzerland; ^3^Graduate School for Cellular and Biomedical Sciences, University of Bern, Bern, Switzerland; ^4^Mymetics SA, Epalinges, Switzerland; ^5^Institute of Anatomy, University of Zürich, Zürich, Switzerland; ^6^Swiss Federal Institute of Intellectual Property, Bern, Switzerland; ^7^School of Veterinary and Life Sciences, Medical and Molecular Sciences, Murdoch University, Perth, WA, Australia; ^8^Telethon Kids Institute, Perth, WA, Australia

**Keywords:** immune modulation, virosomes, liposomes, virus-like particle, respiratory tract, dendritic cell, macrophage

## Abstract

Pulmonary administration of biomimetic nanoparticles loaded with antigen may represent an effective strategy to directly modulate adaptive immune responses in the respiratory tract. Depending on the design, virosomes may not only serve as biomimetic antigen carriers but are also endowed with intrinsic immune-stimulatory properties. We designed fluorescently labeled influenza-derived virosomes and liposome controls coupled to the model antigen ovalbumin to investigate uptake, phenotype changes, and antigen processing by antigen-presenting cells exposed to such particles in different respiratory tract compartments. Both virosomes and liposomes were captured by pulmonary macrophages and dendritic cells alike and induced activation in particle-bearing cells by upregulation of costimulatory markers such as CD40, CD80, CD86, PD-L1, PD-L2, and ICOS-L. Though antigen processing and accumulation of both coupled and soluble antigen was similar between virosomes and liposomes, only ovalbumin-coupled virosomes generated a strong antigen-specific CD4^+^ T cell proliferation. Pulmonary administrated antigen-coupled virosomes therefore effectively induced adaptive immune responses and may be utilized in novel preventive or therapeutic approaches in the respiratory tract.

## Introduction

Immune modulation in the lung may represent a direct approach for treating respiratory disorders such as allergic asthma (1, 2), given that the respiratory tract is readily accessible, making it an ideal target for non-invasive treatments. A dense network of dendritic cells (DCs) ensures mucosal uptake and transport of antigen to lymph nodes for presentation and activation of T cells, as previous studies showed that free antigen is insufficient to induce a strong immune reaction in the respiratory tract (3, 4). In recent years, biomedical nanoparticles for targeted delivery of antigen for vaccinations have been developed (5–7), but to date there is insufficient understanding on how such nanoparticles interact with immune cells in the lung.

Biomimetic nanoparticles such as virosomes and liposomes have already been approved for human use due to their advantageous safety profile and tolerance (8–10). Virosomes may be generated from all enveloped viruses, such as influenza, respiratory syncytial virus (RSV), herpes simplex, human immunodeficiency, rubella, measles, Sendai, West Nile, dengue, yellow fever, or Zika virus (11–16). Virosomes derived from influenza are spherical nanocarriers containing constituents from the influenza virus envelope, hemagglutinin (HA), and neuraminidase (NA) that are incorporated into the phospholipid bilayer. In contrast to liposomes, virosomes are endowed by intrinsic immunogenic properties (11, 12), functioning both as carrier and adjuvant, as they are able to deliver antigens and stimulate immune cells simultaneously (17, 18). Incorporated HA not only provides additional adjuvant function to virosomes but also promotes a rapid cellular uptake by endocytosis (19). HA binds to sialic acid residues that are abundantly expressed on DCs and macrophages (20), thereby triggering highly efficient receptor-mediated uptake.

Within the respiratory tract, immunogens may encounter various immune cells such as macrophages, DCs, and B cells (21). For modulating immune responses, DCs are the preferred target as these are the most potent antigen-presenting cells (APCs). After antigen uptake, DCs undergo so-called maturation, endowing them with the capacity to effectively stimulate CD4^+^ T cells by presenting processed antigen in the context of MHC class II to the T cell receptor. During this maturation process, DCs upregulate surface expression of costimulatory molecules such as CD40, CD80, and CD86 ensuring strong and specific CD4^+^ T cell stimulation. DCs are therefore able to take up, process, and present antigen to naïve CD4^+^ T cells in the lung-draining lymph nodes (LDLNs), thereby regulating T cell responses in the respiratory tract. Viral infection of epithelial cells is first detected by a germline-encoded set of sensors expressed by epithelial cells and innate immune cells [i.e., pattern recognition receptors (PRRs)], which recognize pathogen-associated molecular patterns (PAMPs) originating from the invading viral pathogens [reviewed in Ref. (22)]. PRR sensors include the toll-like receptors, RNA-sensing RIG-I-like receptors, such as retinoic acid-inducible gene I (RIG-I), melanoma differentiation-associated protein 5, and C-type lectin receptors. Despite extensive research, no interaction of influenza virosomes devoid of genetic material was reported to date with PRRs.

In the lung, two major types of migratory conventional DCs have been described, CD11b^+^CD103^−^ and CD11b^−^CD103^+^ DCs (23, 24). CD11b^−^CD103^+^ DCs are localized within the airway epithelium, through which they extend their dendrites to sample antigen from the airway lumen (23), whereas CD11b^+^CD103^−^ DCs are found in the lamina propria and submucosa of conducting airways and sample antigens that have penetrated the epithelium (25, 26).

We have previously shown that both virosomes and liposomes are captured by monocyte-derived DCs and monocyte-derived macrophages in a human triple coculture model, but uptake of virosomes occurred faster, with more virosomes taken up than liposomes in DC monocultures (27). In the current study, we hypothesized that in an *in vivo*-mouse model, virosomes would also constitute an ideal carrier with immune-stimulatory properties that would enable to modulate adaptive pulmonary immune responses. We tested this by applying empty virosomes or liposomes or coupled to OVA with PBS as control intranasally to naïve BALB/c mice and analyzing uptake and phenotypic changes in various respiratory tract compartments. In addition, intracellular processing of antigen and downstream OVA-specific CD4^+^ T cell activation in LDLN was analyzed. To our knowledge, this study represents the first in its field to demonstrate the potential of virosomes for enhanced T cell stimulation in an *in vivo* model for pulmonary application, which highlights these particles as a promising antigen carrier for immune modulation in the respiratory tract.

## Materials and Methods

### Virosome and Liposome Formulation

Influenza virosomes and liposomes were formulated and characterized as previously described in detail (27). Virosome or liposome formulations were either conjugated to the model protein OVA and/or to the fluorochrome Atto647N for detection. All nanocarriers were thoroughly characterized prior to use as recently described (27).

### Mice

A 8- to 12-week-old female BALB/c and DO11.10 T cell receptor-transgenic mice on a BALB/c background were bred specific pathogen-free at the Department of Clinical Research, University of Bern (Bern, Switzerland). Animal work was carried out in accordance with the Swiss Federal Veterinary Office guidelines and was approved by the Cantonal Ethical Committee for Animal Experiments (Amt für Landwirtschaft und Natur des Kantons Bern) under animal experimentation permission number BE71/15.

### Intranasal Administration and Cell Preparation

BALB/c mice were deeply anesthetized for intranasal administration. One hundred (100) microliter total volume of either PBS, virosomes, or liposomes coupled to OVA or DQ-OVA (3 μg total), or empty virosomes or liposomes alone, or coadministered with soluble OVA or DQ-OVA (3 μg total) were applied *via* the nostrils. DQ-OVA was used for degradation and accumulation assays as it consists of OVA bound to a self-quenching fluorescent dye, which upon intracellular degradation releases specific fluorescence (excitation at 505 nm, emission at 515 nm). Accumulated DQ-OVA forming dimers emit fluorescence in a different channel (excitation at 488 nm, emission at 613 nm). Animals were euthanized 24 h after intranasal administration and different lung compartments harvested for determining uptake, trafficking, phenotype and antigen degradation [trachea (T), lung parenchyma (LP), LDLNs, and broncho-alveolar lavage (BAL)], or for T cell proliferation [non-draining lymph nodes (NDLNs) and LDLNs]. Single cell suspensions were prepared as described elsewhere (15, 16). Data for the different experimental groups were obtained from individual animals and at least five independent experiments are shown.

### Flow Cytometry

Digested cells were incubated on ice with FcR block for 10 min followed by viability staining with Fixable Viability Dye eFluor506 (eBioscience, Vienna, Austria) for 30 min on ice. Unless indicated otherwise, antibodies were purchased from eBioscience, and utilized with appropriate isotype controls: CD4-Brilliant Violet 785 (BioLegend, Lucerne, Switzerland), CD69-APC-eFluor 780, DO11.10-PE, CD11c-Brilliant Violet 785 (BioLegend), CD11b-Alexa Fluor 700, MHCII-Brilliant Violet 711 (BioLegend), CD86-Brilliant Violet 605 (BioLegend), CD80-Brilliant Violet 605, CD40-PerCP-eFluor 710, CD8α-PE-eFluor610, PD-L1-PE-Cy7 (BioLegend), PD-L2-FITC, and ICOS-L-PE. Intracellular cytokine staining was performed by using 20 μg/ml Brefeldin A (eBioscience) to stop protein transport. Subsequently, surface marker-stained cells were fixed in a 1% formalin solution followed by intracellular staining with the following antibodies with appropriate isotype control diluted in permeabilization buffer [PBS (Sigma) + 0.1% saponin (Sigma) + 10% FCS (Gibco; Thermo Fisher Scientific, Waltham, MA, USA)]: FoxP3-AlexaFluor 700, IL-4-PE-Cy7, IL-17A-Per-CP-Cy5.5, IFNγ-eFluor450, and IL-9-eFluor660. Acquisition was performed by using a SORP LSRII (BD Biosciences) flow cytometer and data were analyzed by using FlowJo X software (Tree Star, Ashland, OR, USA) and FlowJo9 for T cell proliferation.

### OVA-Specific CD4^+^ T Cell Proliferation in Naïve BALB/c Mice

CD4^+^ T cells from BALB/c DO11.10 mice were negatively selected using Dynabeads untouched mouse CD4 cell kit (Life Technologies, Grand Island, NY, USA). Cells were labeled with carboxyfluorescein succinimidyl ester (CFSE; eBioscience) and 10^7^ cells in 200 μl PBS were injected intravenously into naïve BALB/c mice. After 2 days, virosomes, liposomes, or PBS was administrated intranasally as described above. Three days later, LDLNs and NDLNs were collected and stained for surface markers and intracellular cytokines as mentioned above. Antigen-specific T cell proliferation (CFSE dilution) and cytokine production were measured by flow cytometry and analyzed with FlowJo9 software (TreeStar).

### Statistics

Statistical analyses were conducted using R version 3.2.1 (28). All graphical representations were prepared using the R package ggplot2 (29). Differences in measured frequency and mean fluorescence intensity (MFI) between groups were tested using an ANOVA. Main and interaction effects of OVA (coupled vs soluble OVA) and treatment (liposome, virosome, and controls) were included in the model. Tukey’s honest significant difference *post hoc* test was used to investigate individual paired comparisons. Appropriateness of ANOVA models was verified by residual analysis. No statistically significant difference was detected between “no OVA” (empty nanocarriers) and “coupled OVA” (nanocarriers with coupled OVA) for both virosomes and liposomes in uptake, viability, and phenotype experiments conducted. Therefore, we grouped data utilizing no OVA and coupled OVA virosomes and liposomes to perform the analyses as indicated.

## Results

### Virosome and Liposome Characterization

Virosomes and liposomes were thoroughly characterized for size, homogeneity, particle amount, HA, and OVA content (Table 1) as previously described (27). Briefly, size and homogeneity were measured by dynamic light scattering and by nanoparticle tracking analysis (NTA), routinely providing a diameter of 90–100 nm. NTA analysis yielded approximately 1E+13 particles/ml for all formulations. OVA and HA concentration was determined by SDS-PAGE, Spotblot, and Western Blot, and HA concentrations were selectively reconfirmed by single radial immunodiffusion (data not shown). Both routinely yielded 50 μg/ml HA and 30 μg/ml OVA in the concentrated formulation. For intranasal administration, virosomes and liposomes were employed at a concentration of 3 μg/ml OVA. Limulus amebocyte lysate test of all concentrated formulations showed consistent results below 10.0 EU/ml for endotoxins (data not shown).

**Table 1:** **Particle characterization of virosomes and liposomes**.

Samples	DLS		NTA
	Size ± SD (nm)	PDI ± SD	Size ± SD (nm)
Virosome-Atto647	95.1 ± 1.0	0.035 ± 0.004	76.9 ± 0.4
Virosome-Atto647-OVA	101.0 ± 5.8	0.041 ± 0.010	84.9 ± 0.5
Liposome-Atto647	92.6 ± 2.5	0.027 ± 0.002	79.1 ± 0.8
Liposome-Atto647-OVA	101.3 ± 8.4	0.025 ± 0.015	84.0 ± 0.4

*Virosomes and liposomes coupled either to the fluorochrome Atto647 and/or model protein OVA were analyzed by dynamic light scattering (DLS; Zetasizer Nano S) or by nanoparticle tracking analysis (NTA; NanoSight NS300). Data represent three formulations of virosomes and liposomes with their average modal size (median ± SD, *n* = 3) and the polydispersity index (PDI)*.

### Uptake of Inhaled Virosomes and Liposomes by Respiratory APC Subsets and Trafficking to Lymph Nodes

To detect *in vivo* uptake and trafficking of virosomes or liposomes by APCs situated in different respiratory tract compartments, we harvested T, LP, LDLN, and performed BAL 24 h after intranasal administration of virosomes, liposomes, or PBS control. We gated cells into CD11c^+^MHCII^low^ macrophages, CD11c^+^MHCII^high^CD103^−^CD11b^+^ DCs (CD11b^+^ DCs), and CD11c^+^MHCII^high^CD103^+^CD11b^−^ DCs (CD103^+^ DCs) (23, 30) (Figure S1 in Supplementary Material). As the number of DCs found in BAL was very low, the entire population, mostly CD11b^+^, was analyzed. In LDLNs, cells were subdivided into CD8α^+^ and CD8α^−^, representing resident and migratory DCs, respectively (30, 31). As virosomes and liposomes were suspended in a PBS pH 7.4 solution, we used PBS as uptake control solution (virosome and liposome data were calculated relative to PBS).

**Figure 1:**
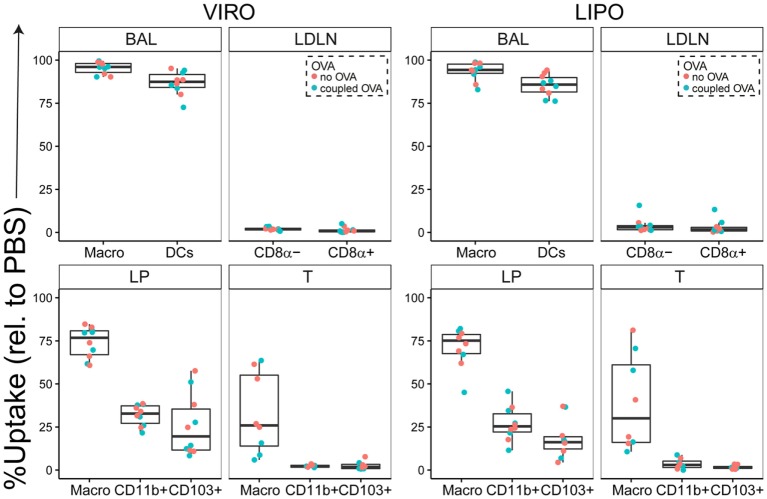
**Uptake of virosomes (VIRO) and liposomes (LIPO) by cells in the respiratory tract**. Liposomes and virosomes without OVA (“no OVA”) or with coupled OVA (“coupled OVA”) were intranasally administered and cells were analyzed for uptake 24 h later in broncho-alveolar lavage (BAL) fluid, trachea (T), lung parenchyma (LP), and lung-draining lymph nodes (LDLNs). Data represent frequency of uptake of five independent experiments. Statistical significance was determined by ANOVA followed by Tukey’s honest significant difference *post hoc* test to investigate individual paired comparisons.

Our results showed that in BAL both virosomes and liposomes, with and without conjugated OVA, were taken up by macrophages and DCs alike, with more than 90% of cells being Atto^+^ (Figure 1). In the LDLN, there was low uptake of both nanocarriers for frequency and MFI (Figure 1; Figure S2 in Supplementary Material). Within the LP, macrophages, and to a lesser extent both subsets of DCs, captured particles, with no difference in uptake detected between virosomes and liposomes (Figure 1; Figure S2 in Supplementary Material). In the trachea, neither CD11b^+^ nor CD103^+^ DCs captured virosomes or liposomes, whereas macrophages showed decent uptake of virosomes and liposomes uptake (Figure 1). In addition, in trachea macrophages also captured more particles per cell (based on MFI intensity) without discriminating between virosomes or liposomes (Figure S2 in Supplementary Material). We further systematically tested cell viability by using a viability dye suitable for detection by flow cytometry. No cell death was detected compared to PBS control for either virosomes or liposomes in any respiratory tract compartment (Figure S3 in Supplementary Material). In conclusion, virosomes and liposomes administered to the respiratory tract were captured most efficiently by macrophages and DCs in BAL, macrophages in trachea and LP, and to a lesser extent DCs in the LP, with low extent of detectable trafficking of either particle to draining lymph nodes 24 h after intranasal administration.

### Antigen-Specific T Cell Stimulation after Exposure to Virosomes or Liposomes

**Figure 2:**
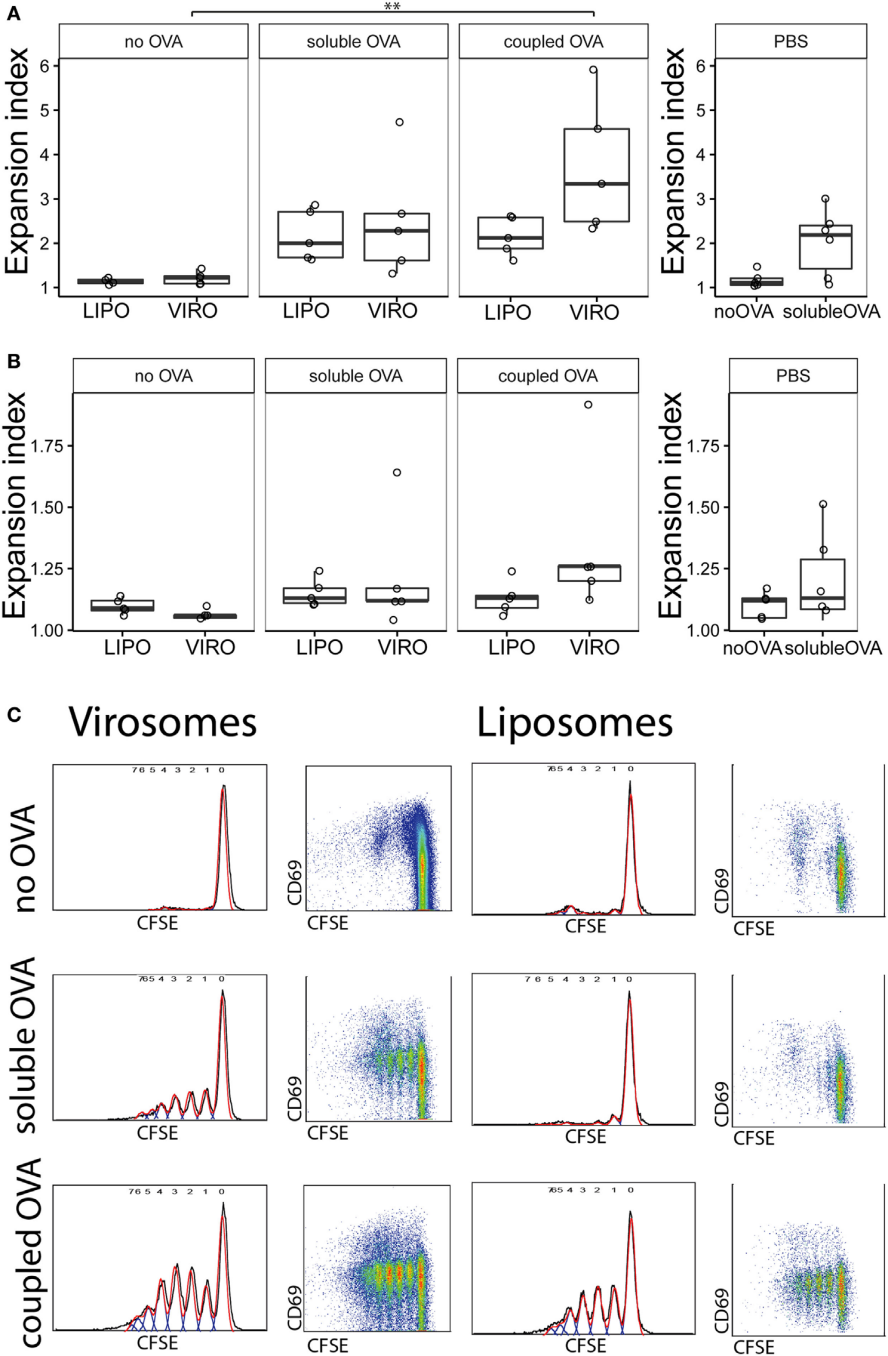
**Measurement of antigen-specific CD4^+^ T cell proliferation by flow cytometry**. Carboxyfluorescein succinimidyl ester (CFSE)-labeled CD4^+^ T cells were injected intravenously in naïve BALB/c mice. After 24 h, virosomes (VIRO), liposomes (LIPO), or PBS was given intranasally. Then 72 h later, lung-draining lymph nodes (LDLNs) **(A)** and non-draining lymph nodes (NDLNs) **(B)** were collected and stained for surface markers. Antigen-specific T cell proliferation (CFSE dilution) was measured by flow cytometry. Panels show the expansion index (EI) of CD4^+^ T cells of six independent experiments. Note the altered *Y*-axis range. Statistical significance was determined by ANOVA followed by Tukey’s honest significant difference *post hoc* test to investigate individual paired comparisons (**p < 0.01). **(C)** FACS gating strategy for T cell proliferation. Cell gating includes forward and sideward scatter for live cells followed by a CD4^+^ DO11.10^+^ gating. Double positive cells were analyzed for CFSE profiles in NDLN and LDLNs to calculate the EI.

We next investigated whether particle-coupled or soluble OVA in combination with empty virosomes or liposomes as adjuvant/nanocarrier would modulate downstream immune response by specifically activating CFSE-labeled, OVA-specific TCR transgenic CD4^+^ T cells as previously described (32). For this purpose, we intranasally administered either empty nanoparticles alone or together with soluble OVA, or nanoparticles coupled with OVA, 2 days after transgenic T cell transfer. Either PBS or PBS together with soluble OVA was employed as controls. As previously described (31), we utilized the FlowJo expansion index (EI) to determine proliferation based on the CFSE dilution profile (Figure S4 in Supplementary Material). The EI is the ratio of final cell count to starting cell count and therefore represents the fold of cellular expansion. OVA coupled to virosomes induced significantly enhanced CD4^+^ T cell proliferation compared to virosomes alone (Figure 2A). Soluble OVA with nanoparticles or PBS induced an approximately twofold increase in EI compared to the no OVA control group. NDLN was used as internal negative control with no significant proliferation detected (Figure 2B). CFSE profiles demonstrated strong T cell proliferation with OVA-coupled virosomes only (Figure 2C). Staining for intracellular cytokines (IFN-γ, IL-17, IL-4, IL-9) and the Treg marker FoxP3 did not reveal any detectable polarization *in vivo* (Figure S5 in Supplementary Material). Taken together, these data show that only antigen coupled to virosomes is able to induce strong antigen-specific T cell response, compared to empty virosomes. Furthermore, we detected no unequivocal evidence of T cell polarization with the markers employed.

### Phenotypic Changes of Respiratory APC Subsets after Exposure to Virosomes and Liposomes

In a next step, we investigated whether stronger T cell proliferation with OVA-coupled virosomes was due to DC activation in LDLN and whether uptake of nanocarriers induced phenotypic changes in respiratory tract macrophage and DC populations. Therefore, respiratory tract compartments (T, LP, LDLN, BAL) were sampled 24 h after intranasal administration of particles and single cells were stained for the surface makers CD40, CD80, CD86, PD-L1, PD-L2, and ICOS-L. Live, single cells were gated as previously described and further subdivided into particle positive (particle^+^) and particle negative (particle^−^) cells based on the Atto fluorescence signal (Figure S1 in Supplementary Material). Without separate analysis of nanocarrier particle positive and negative cells, no phenotypic changes were detected in APC populations after nanocarrier administration (data not shown).

**Figure 3:**
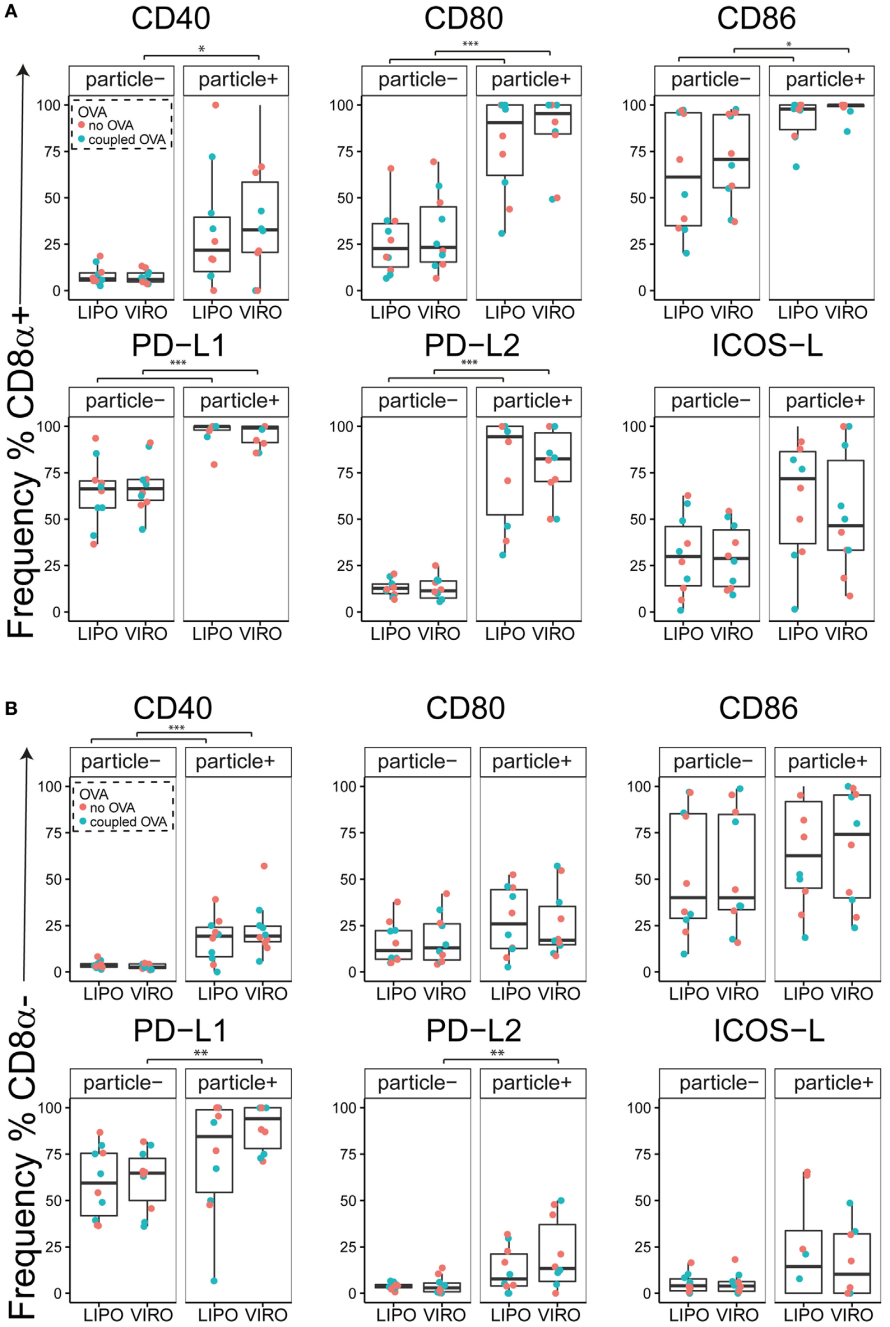
**Expression of surface markers in dendritic cells (DCs) in lung-draining lymph nodes (LDLNs) upon uptake of liposomes (LIPO) and virosomes (VIRO)**. LDLNs were harvested 24 h after intranasal administration of empty liposomes or virosomes (“no OVA”) or with liposomes and virosomes coupled to OVA (“coupled OVA”) or PBS control (not shown). Particle negative (particle^−^) and particle positive (particle^+^) cell populations were analyzed for expression of surface markers CD40, CD80, CD86, PD-L1, PD-L2, and ICOS-L and measured by flow cytometry. Data show frequency (%) of expression of CD8α^+^ resident **(A)** and CD8α^−^ migratory DCs **(B)** and represent five independent experiments. Statistical significance was determined by ANOVA followed by Tukey’s honest significant difference *post hoc* test to investigate individual paired comparisons. *p < 0.05; **p < 0.01; ***p < 0.001.

**Figure 4:**
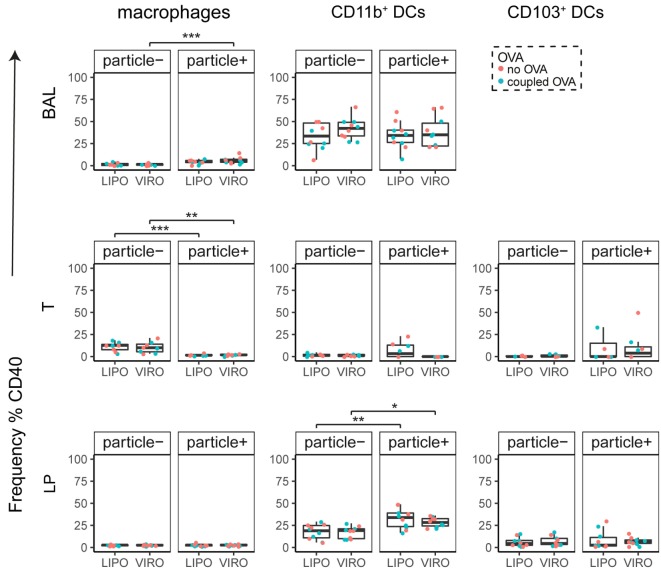
**Expression of surface marker CD40 in pulmonary antigen-presenting cells upon uptake of liposomes (LIPO) and virosomes (VIRO)**. Lung compartments (BAL, broncho-alveolar lavage; T, trachea; LP, lung parenchyma) were harvested 24 h after intranasal administration of empty liposomes or virosomes (“no OVA”) or with liposomes and virosomes coupled to OVA (“coupled OVA”) or PBS control (not shown). Particle negative (particle^−^) and particle positive (particle^+^) cell populations were analyzed for expression of surface marker CD40 measured by flow cytometry and frequency (%) is shown. Data represent five independent experiments. Statistical significance was determined by ANOVA followed by Tukey’s honest significant difference *post hoc* test to investigate individual paired comparisons (*p < 0.05; **p < 0.01; ***p < 0.001).

**Figure 5:**
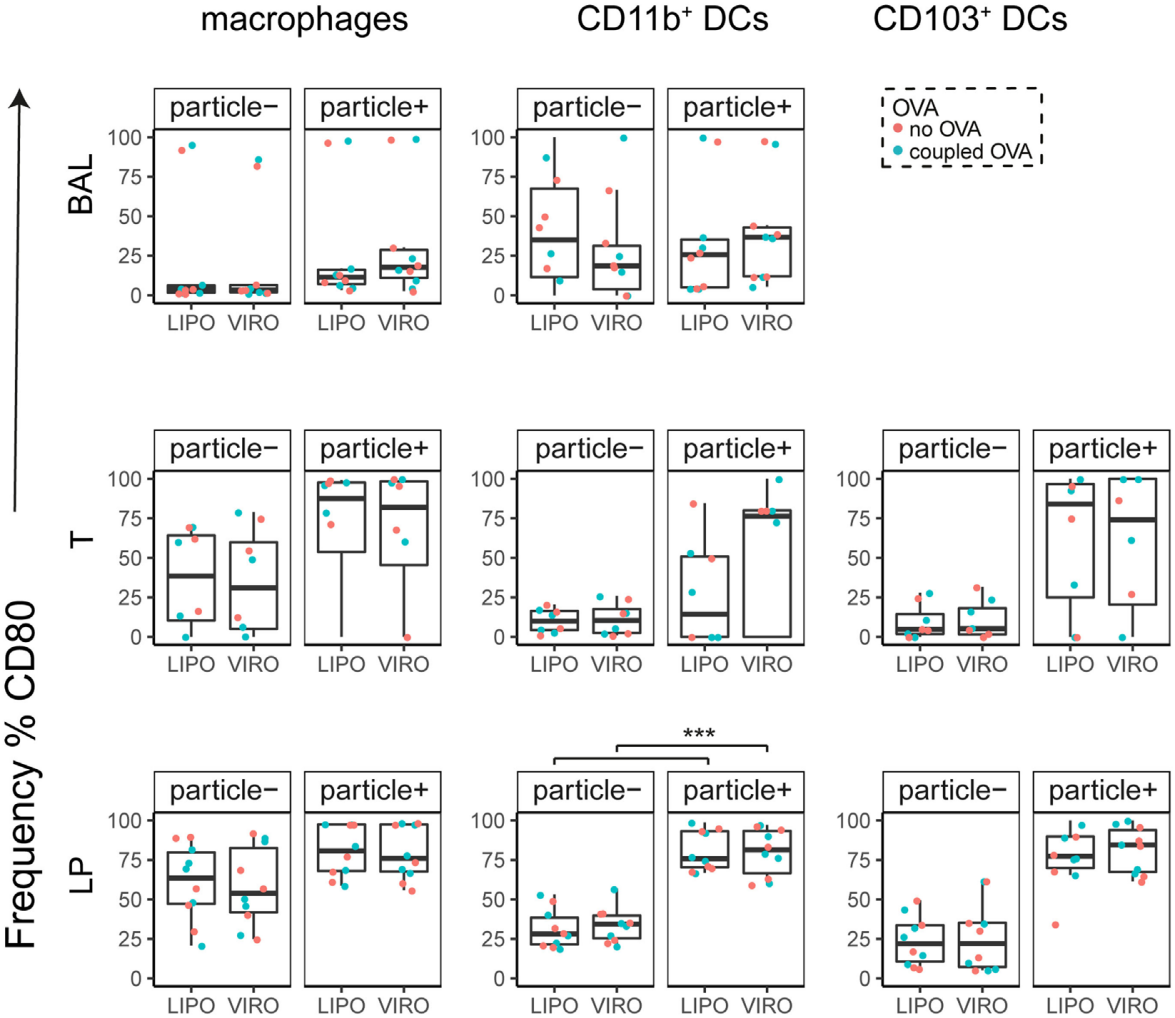
**Expression of surface marker CD80 in pulmonary antigen-presenting cells upon uptake of liposomes (LIPO) and virosomes (VIRO)**. Lung compartments (BAL, broncho-alveolar lavage; T, trachea; LP, lung parenchyma; LDLN, lung-draining lymph nodes) were harvested 24 h after intranasal administration of empty liposomes or virosomes (“no OVA”) or with liposomes and virosomes coupled to OVA (“coupled OVA”) or PBS control (not shown). Particle negative (particle^−^) and particle positive (particle^+^) cell populations were analyzed for expression of surface marker CD80 measured by flow cytometry and frequency (%) is shown. Data represent five independent experiments. Statistical significance was determined by ANOVA followed by Tukey’s honest significant difference *post hoc* test to investigate individual paired comparisons (***p < 0.001).

**Figure 6:**
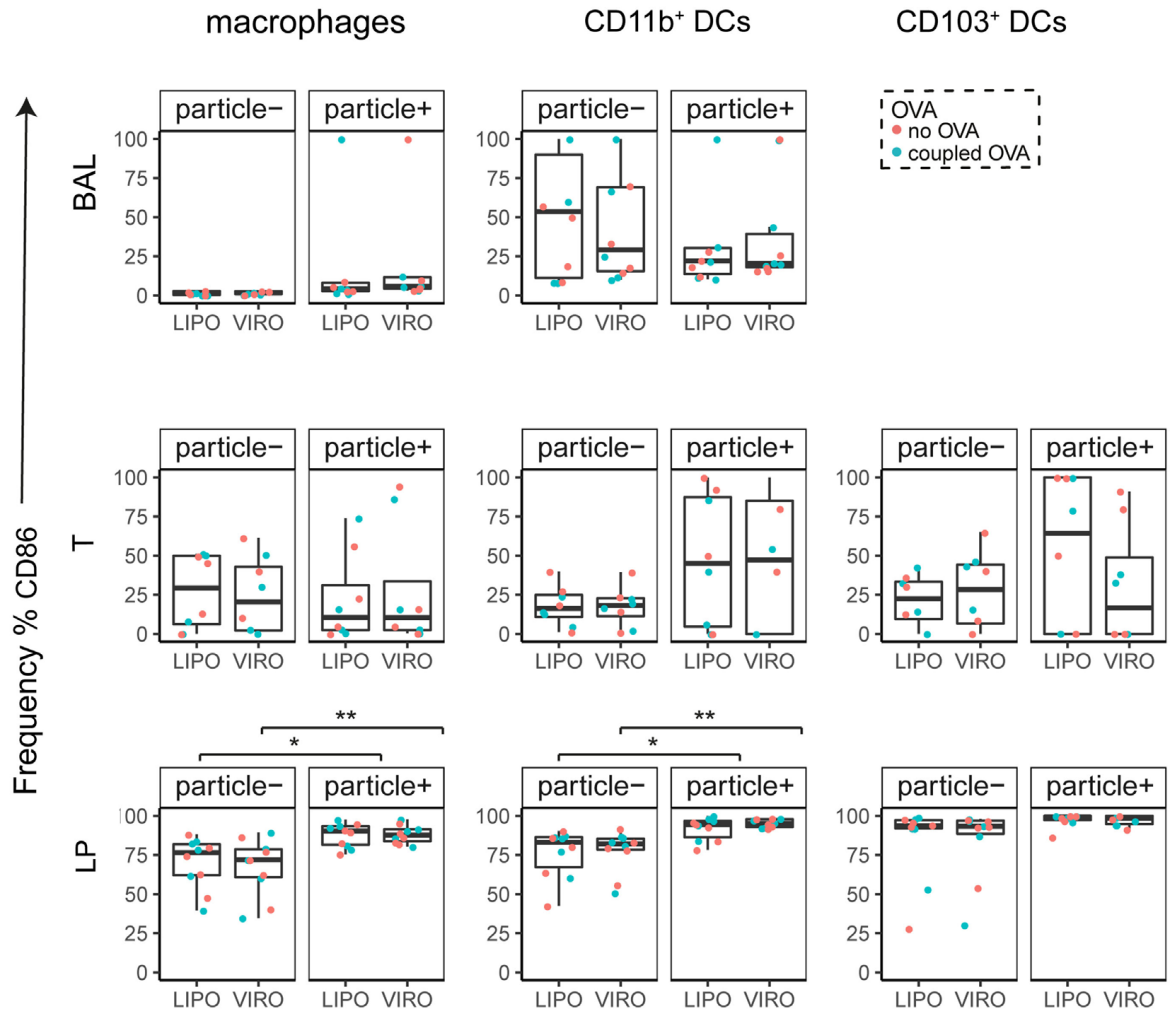
**Expression of surface marker CD86 in pulmonary antigen-presenting cells upon uptake of liposomes (LIPO) and virosomes (VIRO)**. Lung compartments (BAL, broncho-alveolar lavage; T, trachea; LP, lung parenchyma; LDLN, lung-draining lymph nodes) were harvested 24 h after intranasal administration of empty liposomes or virosomes (“no OVA”) or with liposomes and virosomes coupled to OVA (“coupled OVA”) or PBS control (not shown). Particle negative (particle^−^) and particle positive (particle^+^) cell populations were analyzed for expression of surface marker CD86 measured by flow cytometry and frequency (%) is shown. Data represent five independent experiments. Statistical significance was determined by ANOVA followed by Tukey’s honest significant difference *post hoc* test to investigate individual paired comparisons (*p < 0.05; **p < 0.01).

**Figure 7:**
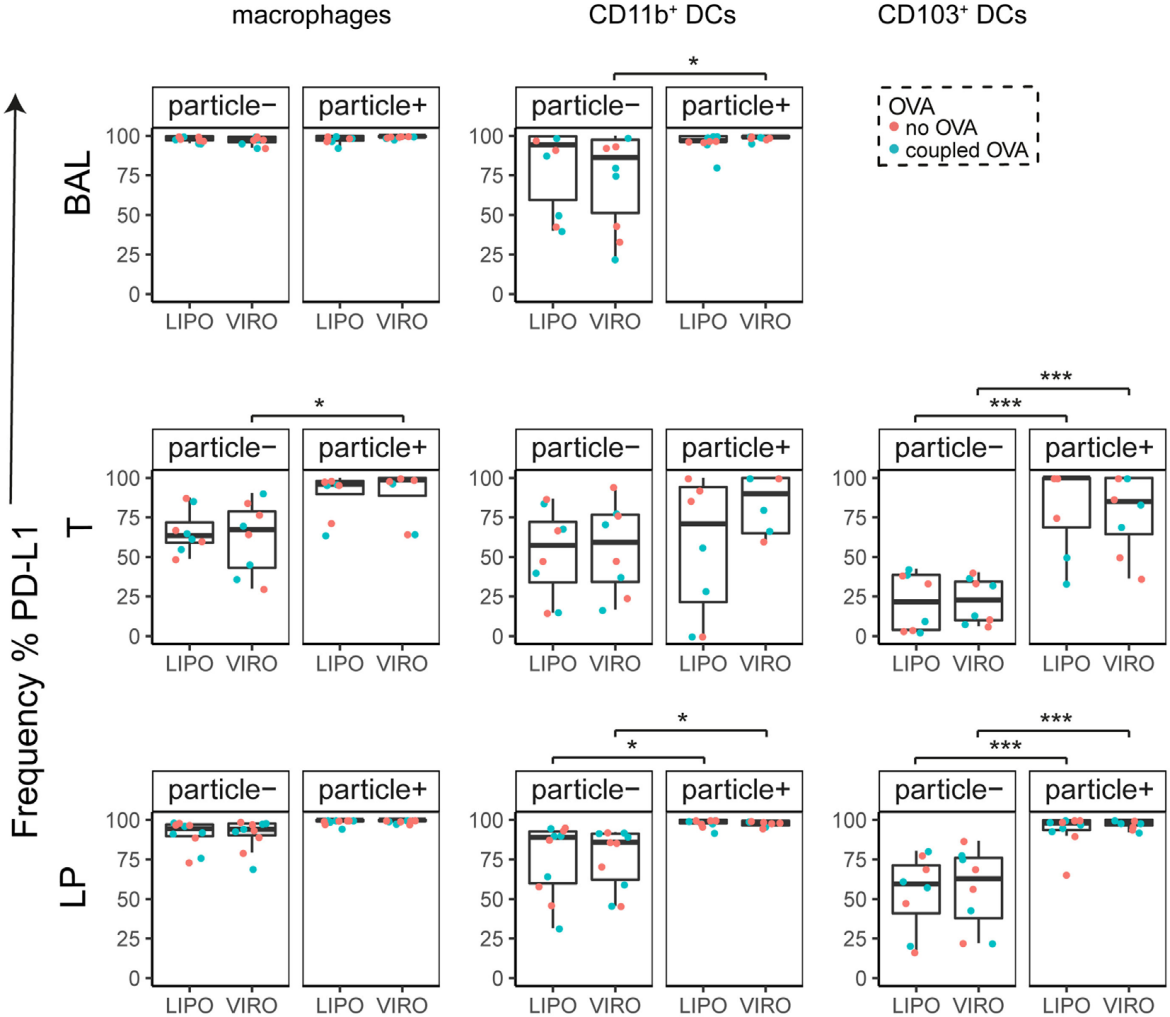
**Expression of surface marker PD-L1 in pulmonary antigen-presenting cells upon uptake of liposomes (LIPO) and virosomes (VIRO)**. Lung compartments (BAL, broncho-alveolar lavage; T, trachea; LP, lung parenchyma; LDLN, lung-draining lymph nodes) were harvested 24 h after intranasal administration of empty liposomes or virosomes (“no OVA”) or with liposomes and virosomes coupled to OVA (“coupled OVA”) or PBS control (not shown). Particle negative (particle^−^) and particle positive (particle^+^) cell populations were analyzed for expression of surface marker PD-L1 measured by flow cytometry and frequency (%) is shown. Data represent five independent experiments. Statistical significance was determined by ANOVA followed by Tukey’s honest significant difference *post hoc* test to investigate individual paired comparisons (*p < 0.05; ***p < 0.001).

**Figure 8:**
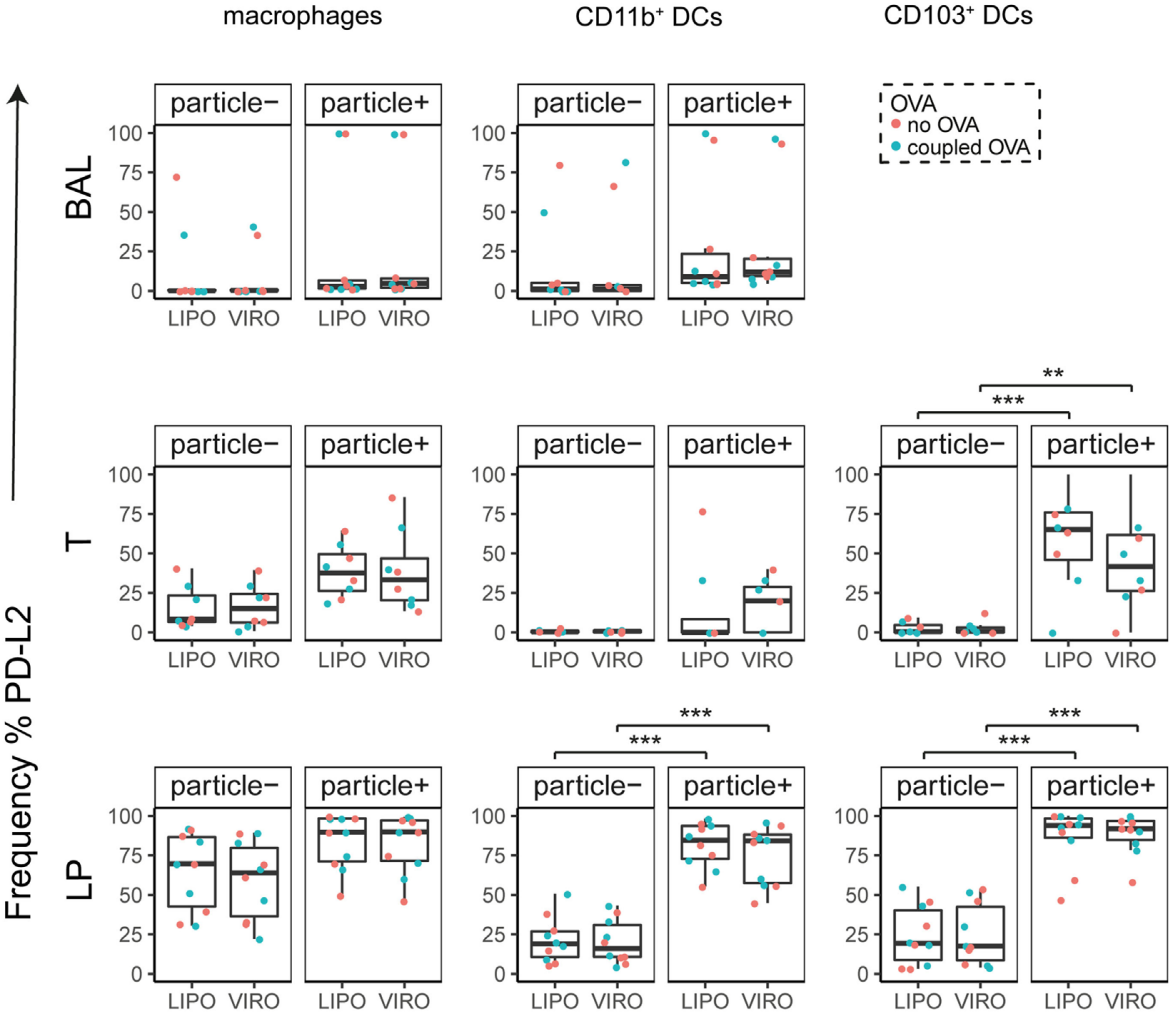
**Expression of surface marker PD-L2 in pulmonary antigen-presenting cells upon uptake of liposomes (LIPO) and virosomes (VIRO)**. Lung compartments (BAL, broncho-alveolar lavage; T, trachea; LP, lung parenchyma; LDLN, lung-draining lymph nodes) were harvested 24 h after intranasal administration of empty liposomes or virosomes (“no OVA”) or with liposomes and virosomes coupled to OVA (“coupled OVA”) or PBS control (not shown). Particle negative (particle^−^) and particle positive (particle^+^) cell populations were analyzed for expression of surface marker PD-L2 measured by flow cytometry and frequency (%) is shown. Data represent five independent experiments. Statistical significance was determined by ANOVA followed by Tukey’s honest significant difference *post hoc* test to investigate individual paired comparisons (**p < 0.01; ***p < 0.001).

**Figure 9:**
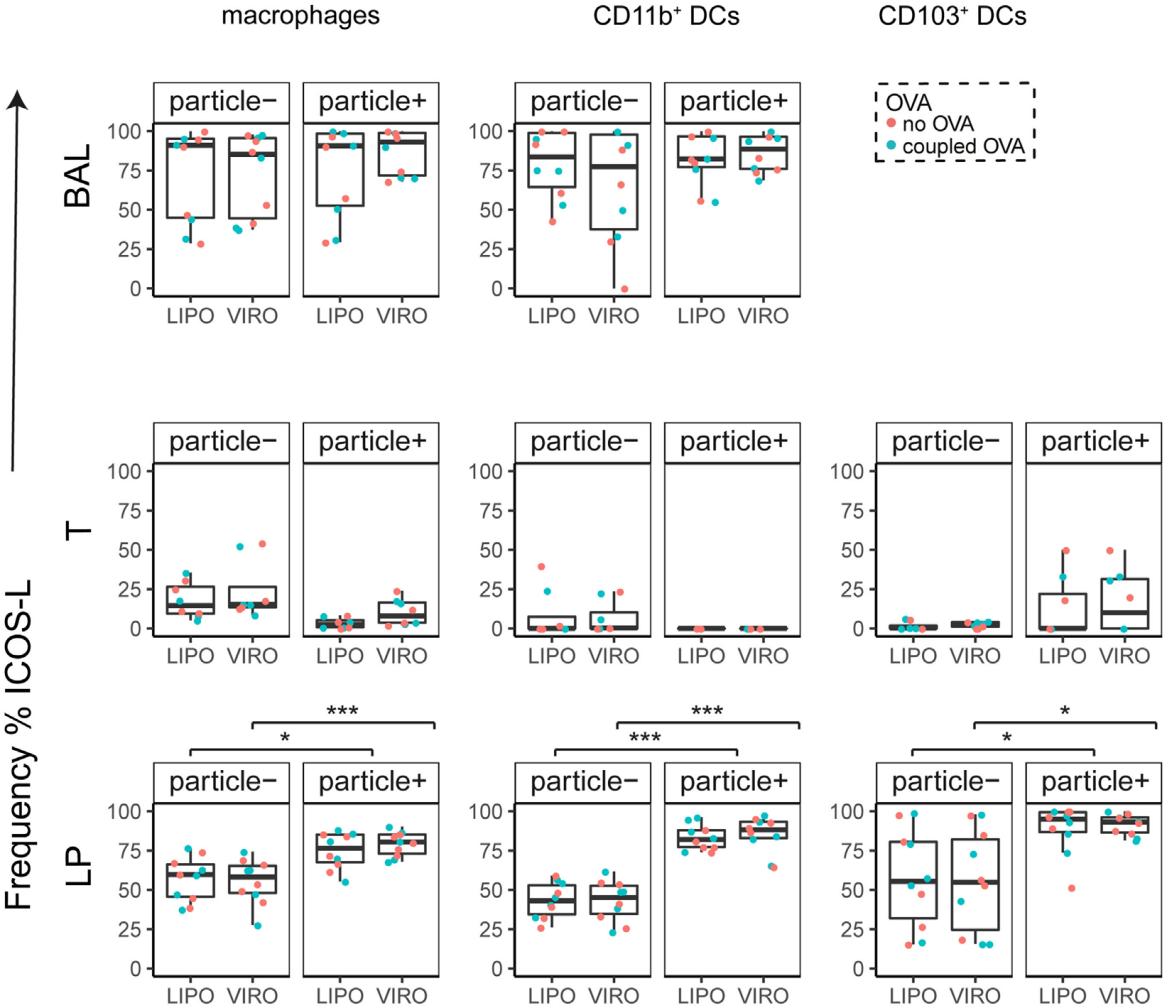
**Expression of surface marker ICOS-L in pulmonary antigen-presenting cells upon uptake of liposomes (LIPO) and virosomes (VIRO)**. Lung compartments (BAL, broncho-alveolar lavage; T, trachea; LP, lung parenchyma; LDLN, lung-draining lymph nodes) were harvested 24 h after intranasal administration of empty liposomes or virosomes (“no OVA”) or with liposomes and virosomes coupled to OVA (“coupled OVA”) or PBS control (not shown). Particle negative (particle^−^) and particle positive (particle^+^) cell populations were analyzed for expression of surface marker ICOS-L measured by flow cytometry and frequency (%) is shown. Data represent five independent experiments. Statistical significance was determined by ANOVA followed by Tukey’s honest significant difference *post hoc* test to investigate individual paired comparisons (*p < 0.05; ***p < 0.001).

Interestingly, CD8α^+^ resident DCs in LDLN significantly upregulated most costimulatory markers after uptake of particles (Figure 3A, particle^+^ DCs), and significant changes in MFI for CD40, CD80, and ICOS-L for virosomes in CD8α^+^ cells occurred (Figure S6A in Supplementary Material). CD8α^−^ migratory DCs in LDLN significantly upregulated CD40, PD-L1, and PD-L2 (Figure 3B) and MFI was increased for CD40 (Figure S6B in Supplementary Material). Furthermore, a low but significant increase in CD40 expression frequency in both macrophages in BAL and trachea and CD11b^+^ DCs in LP occurred (Figure 4), but no changes were detectable for the MFI (Figure S7A in Supplementary Material). CD80 expression significantly increased upon uptake of particles in CD11b^+^ DC cell populations in LP for both frequency (Figure 5) and MFI (Figure S7B in Supplementary Material), whereas CD86 expression was increased after particle uptake for both macrophages and CD11b^+^ DCs in LP (Figure 6). A significantly higher proportion of CD103^+^ DC expressed PD-L1 in both trachea and LP whereas CD11b^+^ DCs upregulated PD-L1 in LP and BAL after particle uptake (Figure 7). Following particle uptake, CD11b^+^ DCs from BAL and LP showed greater expression of PD-L1, as did macrophages and CD103^+^ in LP (MFI; Figure S7D in Supplementary Material). An increased proportion of PD-L2 positive cells was found in the CD11b^+^ DCs from LP, as well in CD103^+^ DCs from trachea and LP (Figure 8) following particle uptake, whereas for MFI we detected significant change for macrophages, CD11b^+^ and CD103^+^ cells in LP, and CD11b^+^ DCs in T (Figure S7E in Supplementary Material). Similarly, for ICOS-L upregulation, an enhanced proportion of cells was seen in particle-bearing macrophages, CD11b^+^ and CD103^+^ DCs from LP (Figure 9), and an increased MFI in macrophages from BAL and DCs in LP (Figure S7F in Supplementary Material).

Our data therefore indicate that particle uptake was associated with phenotypic changes, regardless of whether liposomes or virosomes were administered. Taken together we could detect activation of DCs in LDLN (CD40, CD80, CD86, PD-L1, PD-L2), BAL (CD40, PD-L1), trachea (CD40, PD-L1, PD-L2), and LP (CD40, CD80, CD86, PD-L1, PD-L2, ICOS-L), yet with no differences seen between liposomes and virosomes.

### Antigen Degradation by APCs after Exposure to Virosomes and Liposomes

**Figure 10:**
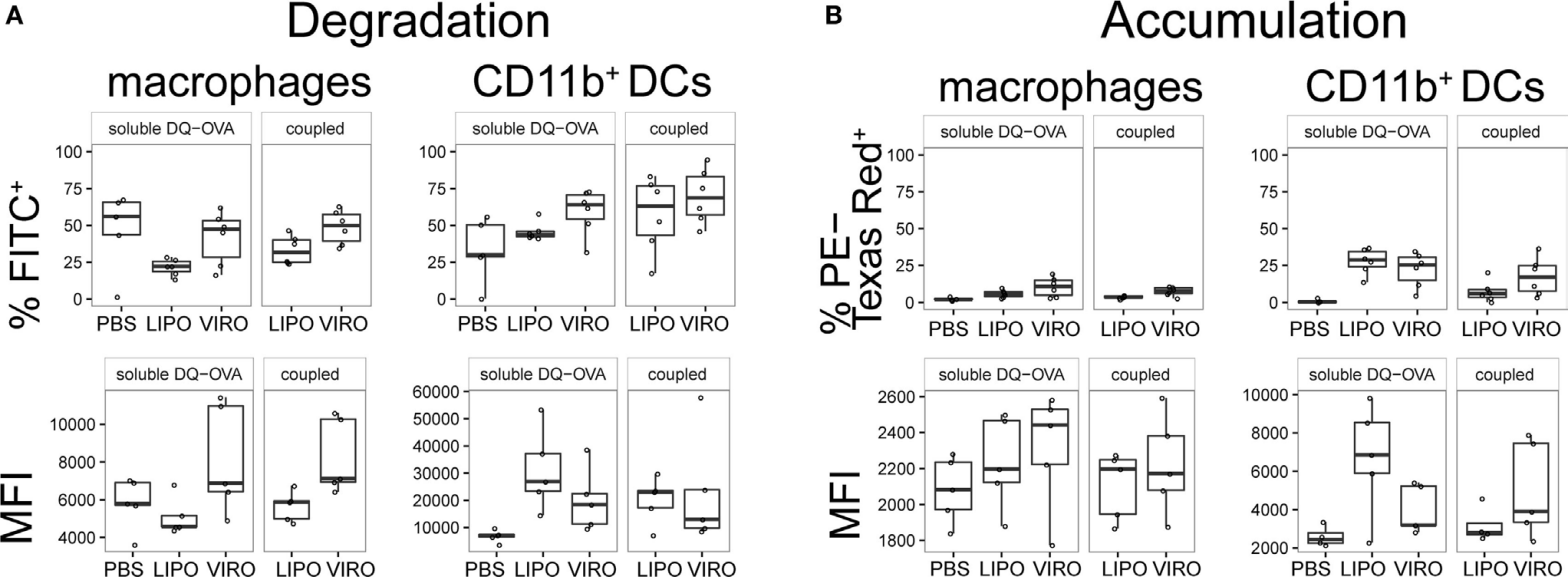
**Antigen degradation and accumulation in BAL**. BAL was collected 24 h after intranasal administration of empty liposomes, virosomes, or PBS with soluble DQ-OVA (“soluble DQ-OVA”) or with liposomes and virosomes coupled to DQ-OVA (“coupled DQ-OVA”). Antigen degradation **(A)** and accumulation **(B)** was analyzed by measuring released fluorochrome signal from self-quenching DQ-OVA by flow cytometry. Panels show frequency (%) and MFI of six independent experiments. Statistical significance was determined by ANOVA followed by Tukey’s honest significant difference *post hoc* test to investigate individual paired comparisons.

In a next step, we measured whether uptake of nanocarriers was associated with alterations in antigen processing in APC subsets. For this analysis, DQ-OVA was coupled to virosomes and liposomes, or administered in its soluble form simultaneously with empty nanocarriers intranasally. DQ-OVA consists of OVA bound to a self-quenching fluorescent dye, which upon intracellular degradation releases specific fluorescence (excitation at 505 nm, emission at 515 nm). Accumulated DQ-OVA on the other hand, forms dimers between dyes and emits fluorescent signal in a different channel (excitation at 488 nm, emission at 613 nm). Controls were performed with PBS alone or supplemented with soluble DQ-OVA to determine the possible adjuvant effect of virosomes or liposomes. In BAL, antigen degradation and accumulation was detectable both in macrophages and DCs, but with no significant differences seen between liposomes and virosomes (Figure 10). Overall we observed a low number of cells that degraded DQ-OVA with no difference detected between soluble or coupled DQ-OVA (Figure S8 in Supplementary Material) and antigen accumulation was very low in other respiratory tract compartments (Figure S9 in Supplementary Material).

## Discussion

The ease of access of the respiratory tract makes it an attractive target organ for the administration of immunomodulatory biomimetic antigen nanocarriers. These may elicit their effects through interactions with a tightly enmeshed network of DCs that will capture and traffic nanocarriers to regional lymph nodes for specific T cell activation. Pulmonary administration of biomimetic nanoparticles such as virosomes or liposomes coupled to antigen may therefore represent a novel effective strategy to directly modulate adaptive immune responses in the respiratory tract. Virosomes not only serve as antigen carriers but are also endowed with intrinsic immune-stimulatory properties, as virosomes themselves are able to activate APCs and enhance uptake and processing of antigen (33, 34). Immune-stimulatory properties derive from incorporated viral envelope proteins HA and NA, as recombinant influenza HA alone is able to induce DC activation (35–37). Activation of DCs by virosomes induced a Th1 type of cytokine profile (34). Additionally, surface features of virosomes are indistinguishable from those of the parental virus they derive from. Repetitive patterns of such viral surface structures render virosomes highly immunogenic by mimicking damage-associated molecular patterns and PAMPs, eventually activating DCs through interaction with PRR (38–40).

There is still insufficient data available on the fate of virosomes and liposomes administered to the respiratory tract, as most clinical trials administered virosomes either intramuscular or intradermal. Virosomes delivered intranasally provided protection against influenza that was comparable to the intramuscular application (36, 37). Additionally, virosomes provided both a systemic and mucosal immune response upon intranasal delivery of DNA as compared to the intradermal route of administration (41). *In vivo* intranasal administration of a RSV-derived virosome vaccine induced high antibody titers and provided complete protection from RSV infection (42). Virosomes derived from respiratory viruses such as influenza and RSV may not only offer protective immunity but may potentially also provide a novel strategy for therapeutic immune modulation in the respiratory tract. In this study, we investigated the interaction of such nanocarriers with APCs in the respiratory tract, using an *in vivo* model to analyze uptake, phenotype and antigen processing capacity by macrophages, and DCs in different respiratory tract compartments. We also measured *in vivo* antigen-specific T cell stimulation by virosome- and liposome-bound antigen. Though our data show that both virosomes and liposomes are internalized by DCs, inducing their activation, only virosome-bound antigen generated a robust-specific CD4^+^ T cell proliferation in draining lymph nodes.

In the current study, both virosomes and liposomes were captured by respiratory tract APCs, but the extent of uptake depended on localization of APCs within the respiratory tract compartments. APCs in the alveolar space (BAL), through their prolonged interaction, were highly positive for the administered nanocarriers. Though tracheal APCs are an initial cell population to encounter virosomes and liposomes, the exposure is limited since particulate antigen deposited in larger airways is rapidly cleared by the mucociliary escalator. This contrasts with the clearance in the alveolar gas-exchange regions, where clearance by alveolar macrophages can take up to 24 h (43). Persistent antigen in the alveolar space due to slower clearance may explain the stronger and most consistent uptake signal detected in this compartment. By contrast, the levels of virosomes and liposomes were very low 24 h after intranasal delivery, the optimal time point for active delivery of inhaled antigens to DLN. By differential staining for CD8α, we were able to distinguish active transport from the airways by CD8α^−^ DC from passive drainage to the LDLN and uptake by CD8α^+^ DCs. Migratory CD8α^−^ DCs traffic antigen to LDLN, inducing antigen-specific T cell activation (44). By contrast, antigen that passively drains to the LDLN is primarily taken up by resident CD8α^+^ DCs. Due to low cell numbers and low particle signal obtained from lymph node cell suspensions, and the technically demanding FACS analysis, we failed to detect measureable levels of either soluble or coupled OVA that had drained to the LDLN or was actively transported by migrating DCs. The reason for this is unclear, but presumably relates to the preferential targeting of particles to non-migratory macrophages in the airways and LP. Our group previously showed that different types of particles delivered *via* the pulmonary route are primarily taken up by alveolar macrophages (31, 32), a finding which we also confirmed for both virosomes and liposomes. Alveolar macrophages are the first line of defense and hence play an important role in clearing apoptotic cells, debris as well as inhaled pathogens and particles to maintain lung homeostasis (45, 46). Contrary to DCs, lung macrophages are unable to efficiently stimulate T cells, and they do not migrate to local draining lymph nodes following antigen uptake. Enhanced uptake of particles by alveolar macrophages compared to pulmonary DC has also been shown by Jakubzick et al. (47). In this study, naïve C57BL/6 mice were instilled intranasally with 500 nm polystyrene particles. In accordance with our findings, the majority of particles were found inside alveolar macrophages with very few particles transported to the LDLNs by pulmonary DCs 2 days after instillation. However, when macrophages were depleted with clodronate integrated in liposomes, markedly greater numbers of DCs were recruited into the alveolar space and particle transport to the LDLNs was boosted by around 20-fold. The authors concluded a macrophage-mediated suppression of airway DC function by the modulation of DC recruitment to the airways. Pulmonary DCs offer an ideal target to modulate adaptive immune responses (30, 48). Enhanced targeting of DCs in the respiratory tract may be achieved by binding of DC-specific ligands such as anti-DEC205 or anti-DC-SIGN onto the lipid bilayer (49, 50). Also, previous studies showed that liposomes bound to IgG increased binding and uptake by DCs *via* FcγR (51). Virosomes and liposomes may also be modified by either coupling antigen of interest onto the surface or incorporating it into the lumen (52). For example, a “self” recognition peptide of CD47, when coupled to nanoparticles, was recently shown to avoid phagocytosis by macrophages as it is recognized as self, therefore prolonging nanoparticle circulation and hence the duration for drug delivery (53). Combining CD47 and a DC-specific ligand on virosomes or liposomes may potentially increase uptake by DCs and reduce phagocytosis by macrophages.

Though we detected only a weak signal of particle^+^ DCs in LDLN, OVA-coupled virosomes generated a significantly increased naïve OVA-specific CD4^+^ T cell proliferation. Soluble OVA in combination with liposomes, virosomes, or PBS generated approximately a twofold increase of CD4^+^ T cell proliferation. Virosome-coupled antigen induced a significant increase of CD4^+^ T cell proliferation at a low OVA concentration (3 μg total), compared to previous results in our group using gold or polystyrene particles where 50 μg of OVA was necessary to induce a comparable CD4^+^ T cell response (31, 32). This is also in line with a previous study, showing that a dose as low as 0.75 μg antigen (OVA) in combination with virosomes induced a strong cytotoxic T cell (CTL) response *in vivo* following intramuscular, intraperitoneal, or subcutaneous injection (18), contrasting with other two studies reporting that between 30 and 130 times more OVA is required to induce CTL when using liposomes (54). More importantly, successful T and B cell stimulation with low doses of peptides associated virosomes was also demonstrated in clinical studies (55, 56). These findings show that virosomes function both as an antigen carrier and adjuvant when applied systemically, and we have demonstrated a similar effect when virosomes were delivered locally to the lungs. Enhanced T cell responses to virosome-coupled antigen may first be due to the intact particulate characteristic of the antigen–virosome complex. As an example, employing gold nanoparticles coated with an aminated polymer shell, we have previously shown that only the particle–shell complex, but not the polymer alone was able to induce enhanced OVA-specific T cell proliferation in BALB/c mice (32). Second, the combination of the antigenic viral compounds HA and NA may modulate antigen presentation in the LDLN. The virosome surface is responsible for a stronger immune stimulation, probably due to the addition of HA and NA, generating not only OVA-specific T cells but also HA-specific ones and therefore enhancing the overall immune response. Pre-existing immunity to influenza virus has no negative effects in humans (57, 58) but shows to be an advantage as both antigen-specific and influenza-specific T helper cells stimulate B cells and CTLs for strong immune response (34, 59). Mouse studies revealed that immunization with influenza virus resulted in higher antibody titers when administered with virosomes as adjuvants (60).

To investigate why only OVA-coupled virosomes induce strong and specific T cell response, we stained lung APCs for different surface markers and analyzed phenotype and costimulation marker expression by flow cytometry. Enhanced surface expression of costimulatory molecules like CD40, CD80, and CD86 is essential for induction of a strong and specific T cell response (61). Therefore, we measured expression levels of these markers and in addition investigated the expression of PD-L1, PD-L2, and ICOS-L, interesting targets for induction of tolerance (62, 63). PD-L1 and PD-L2 are both ligands for programmed death-1 (PD-1), expressed on activated T cells regulating T cell activation and tolerance. However, PD-L1 and PD-L2 expression remains controversial, as they can have both stimulatory and inhibitory effects on T cells (64–67), as was seen in studies using OVA-specific T cell proliferation assays showing that overexpression or blocking of either of these two ligands interfered with T cell activation and proliferation (64, 68). Others found that blocking PD-L1 and PD-L2 resulted in increased T cell proliferation (69–71). In allergic diseases like asthma, the role of PD-L1 and PD-L2 is also still unclear as it was shown that both ligands have opposing roles in the regulation of airway hyperreactivity (AHR) (63). Akbari et al. demonstrated that PD-L2-knockout mice had increased AHR and inflammation (72), which was confirmed by a study that blocked PD-L2 during antigen challenge inducing increased AHR (73, 74). On the other hand, it was shown that PD-L1 deficiency had a positive effect on AHR and inflammation (63). A recent study performed by McAlees et al., however, showed that blocking the PD-L1/PD-1 pathway in an allergic asthma mouse model resulted in enhanced AHR by induction of Th17 cells in a mild AHR animal model (75). The inducible costimulatory ligand, ICOS-L, was also shown to play a role in an experimental asthma model as ICOS-deficient mice showed decreased Th2 response (76, 77) and ICOS–ICOS-L proved to be essential for the induction of T regulatory cells (78). In allergic rhinitis and asthma patients, it was demonstrated that ICOS-L expression on mDCs is impaired, contributing to the Th2-type immune response (79).

In our study, we observed phenotypic changes of DCs only in particle-bearing (particle^+^) cell populations, indicating that uptake of particles is essential for upregulation of phenotypic markers in DCs, but not in macrophages. Specifically, we observed upregulation of classical costimulatory markers such as CD40, CD80, and CD86 on both macrophages and DCs, indicating a moderate activation of DCs enabling T cell stimulation. On the other hand, we also observed even stronger upregulation of PD-L1 and PD-L2 in macrophages and DCs, which, as already stated, can have different effects on the polarization and tolerogenic state of T cells. ICOS-L is enhanced in LP macrophages and DCs alike, indicating a potential for strong T cell stimulation and polarization away from Th2, as mentioned previously. Overall, this indicates that particle uptake is associated with DC activation and small amounts of particles were sufficient to trigger measurable phenotypic changes. From these data, we conclude that uptake of both virosomes and liposomes activated DCs, but the degree of activation primarily depended on the anatomical location, a finding that is consistent with previous data from our group obtained from *in vivo* studies using gold nanoparticles that showed CD40 and CD86 upregulation upon particle uptake (32). Differences in the degree of activation seen in CD103^+^ and CD11b^+^ DCs may be explained by the fact that both subsets have distinct functional properties (80), with CD103^+^ DCs localized along the airways that are able to extend their dendrites through the epithelial tight junctions and therefore sample inhaled antigen from the airway lumen (23, 81). Additionally, it is known that this cell type is able to cross-present antigen to CD8^+^ T cells (81–83). CD11b^+^ DCs on the other hand are assumed to be located mostly in the submucosa of the conducting airways and therefore only sample antigen that has crossed the epithelial layer. In our study, we did not detect any differences in uptake between these two DC subsets, possibly indicating similar access and uptake of virosomes and liposomes from the airway lumen and the submucosa.

To understand whether uptake of OVA-coupled virosomes or liposomes induced functional changes in macrophages and DCs, we measured the capacity of DCs to degrade coupled or soluble antigen. Self-quenching DQ-OVA was coupled to either virosomes or liposomes, or administered soluble together with empty particles. No difference in antigen degradation occurred between virosomes or liposomes, delivering either coupled or soluble DQ-OVA. The strongest degradation and accumulation signal was detected in BAL macrophages and DCs that paralleled the strong uptake of nanoparticles seen in cell populations of this compartment. Despite comparable antigen degradation and activation seen after treatment with OVA-coupled liposomes or soluble OVA co-administered with empty virosomes or liposomes, no significant increase in CD4^+^ T cell proliferation compared to empty virosomes or liposomes was observed in these experimental groups.

To our knowledge, these findings are unique, showing that very low antigen concentrations bound to virosomes and administered to the respiratory tract are sufficient to generate a robust antigen-specific CD4^+^ T cell response in LDLN. This may have consequences if the pulmonary route is utilized for novel vaccination strategies, as concentration of virosome-bound antigen may be kept to a minimum, yet still generate a robust adaptive immune response. Though several studies showed a Th1 polarization after virosome stimulation (34, 84, 85), this did not occur in our *in vivo* model. Specifically targeting DCs to modulate CD4^+^ T cell responses may represent a promising approach to treat allergic asthma where an enhanced Th2 response frequently leads to IgE production (IL-4), eosinophilia (IL-5), mast cell activation (IL-9), and AHR (IL-13) (86, 87). Specific immune therapy, either subcutaneous or sublingual, is available for selected patients, but treatments last several years and are of variable efficacy (88–90). Pulmonary administered virosome-bound low-dose antigen may overcome such limitations with its potential to specifically modulate innate CD4^+^-dependent immune responses, without causing excessive inflammatory responses that may jeopardize vital gaseous exchange in the lung.

In summary, we have demonstrated that intranasally administered virosomes and liposomes are taken up by macrophages and DCs in the respiratory tract and induce DC activation. There was no detectable difference in antigen processing or accumulation, but only OVA-coupled virosomes generated a specific and robust CD4^+^ T cell activation not seen with OVA-coupled liposomes or soluble OVA co-administered with virosomes or liposomes. Pulmonary administered antigen-bound virosomes may therefore provide an attractive approach to specifically and safely modulate adaptive immune responses in the respiratory tract, either to generate a protective immunity through vaccination, or as an approach for immune therapy in allergic asthma.

## Author Contributions

Study design: RB, FB, and CG. Data curation: RB. Formal analysis: RB and RD. Visualization: RD. Supervision: MA, FB, and CG. Funding: CG. Writing original draft: RB. Writing review and editing: RB, MA, PS, FB, and CG.

## Conflict of Interest Statement

The authors have no other affiliations or financial involvement with any organization or entity with a financial interest related to this study.
