# Supplementary material for: Pulmonary Delivery of Virosome-Bound Antigen Enhances Antigen-Specific CD4+ T Cell Proliferation Compared to Liposome-Bound or Soluble Antigen
Source: Front Immunol. 2017 Apr 7;8:359. doi: 10.3389/fimmu.2017.00359 (PMC5383731; doi:10.3389/fimmu.2017.00359)
Supplement: Supplementary file 1 [file Data_Sheet_1.PDF]

Supplementary Material

Pulmonary Delivery of Virosome-Bound Antigen Induces Antigen-Specific CD4+ T Cell Proliferation

Rebecca A.M. Blom[1,2,3], Mario Amacker[4], R. Maarten van Dijk[5], Christian Moser[6], Philip A. Stumbles[7,8], Fabian Blank[1,2,*,#], Christophe von Garnier[1,2,#]

[1]Department of Pulmonary Medicine, Inselspital, Bern University Hospital, University of Bern, Bern, Switzerland.
[2]Department of Clinical Research, University of Bern, Bern, Switzerland
[3]Graduate School for Cellular and Biomedical Sciences, University of Bern, Bern, Switzerland.
[4]Mymetics SA, Epalinges, Switzerland.
[5]Institute of Anatomy, University of Zürich, Zürich, Switzerland.
[6]Swiss Federal Institute of Intellectual Property, Bern, Switzerland.
[7]School of Veterinary and Life Sciences, Medical and Molecular Sciences, Murdoch University, Perth, Australia
[8]Telethon Kids Institute, Perth, Australia

[#]CvG and FB contributed equally to this work

***Correspondence:** Dr. Fabian Blank, Respiratory Medicine, Department of Clinical Research, Murtenstrasse 50, 3008 Bern, Switzerland, e-mail: fabian.blank@dkf.unibe.ch, Tel: +41 31 632 76 34, Fax +41 31 632 75 94

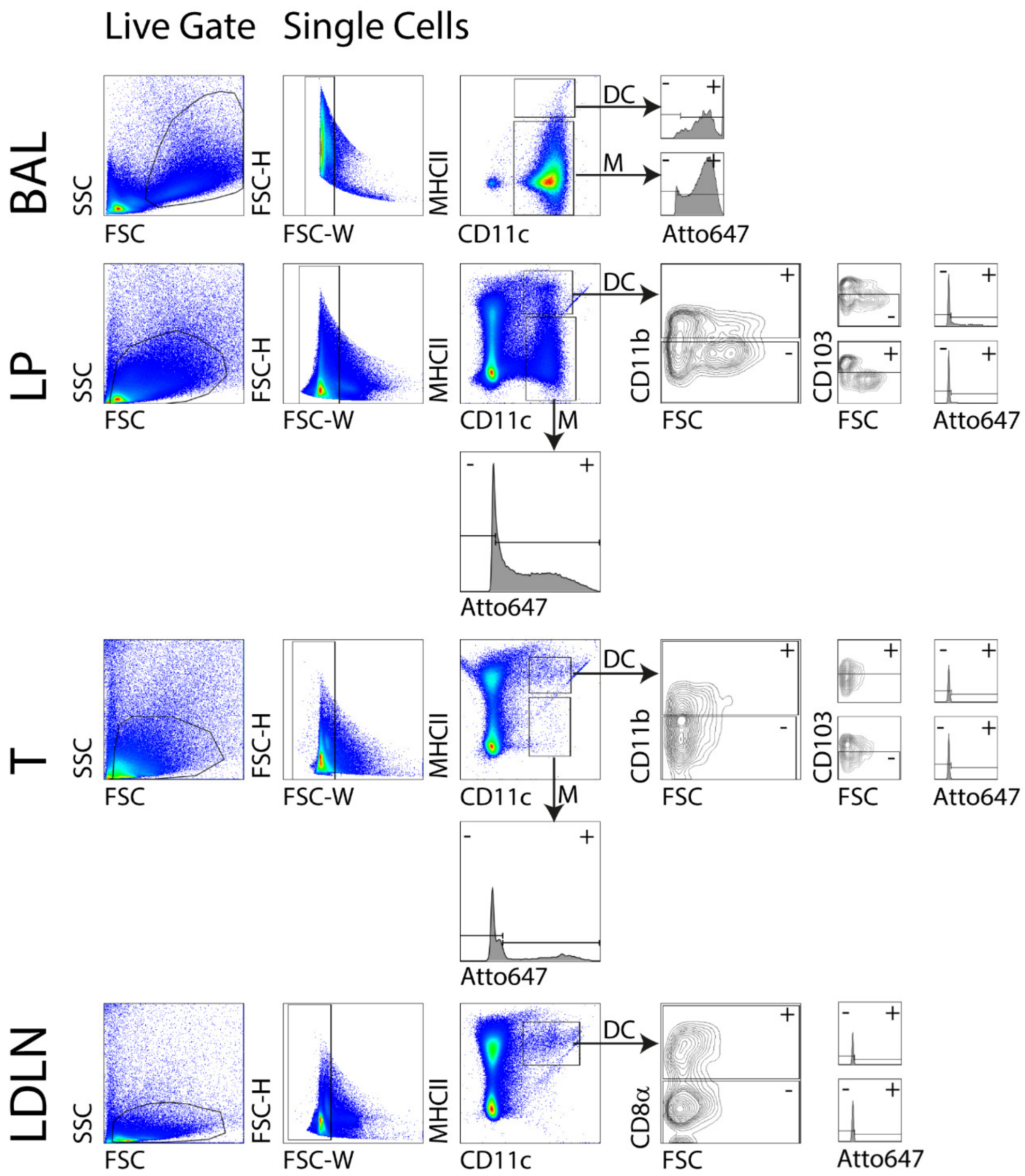

Supplementary Figure 1: FACS gating strategy for APC populations in different respiratory tract compartments. Cells of interest were gated according to forward (FSC) and sideward (SSC) scatter followed by a single cells gate (FSC-H/FSC-W). Dendritic cells (DCs) and macrophages (M) were gated according to CD11c and MHC class II (MHCII) expression. In broncho-alveolar lavage (BAL) these cells were subdivided into particle+ and particle- cells according to Atto647signal. In lung parenchyma (LP) and trachea (T), DCs were first subdivided into CD11b positive or negative cells and further into CD103 positive or negative cells. $CD11b^{+}CD103^{-}$ and $CD11b^{-}CD103^{+}$ DCs were then analyzed for particle positive or negative subsets according to Atto647 signal. In lung-draining lymph nodes (LDLN) DCs were divided into migratory or resident cells according to CD8α expression.

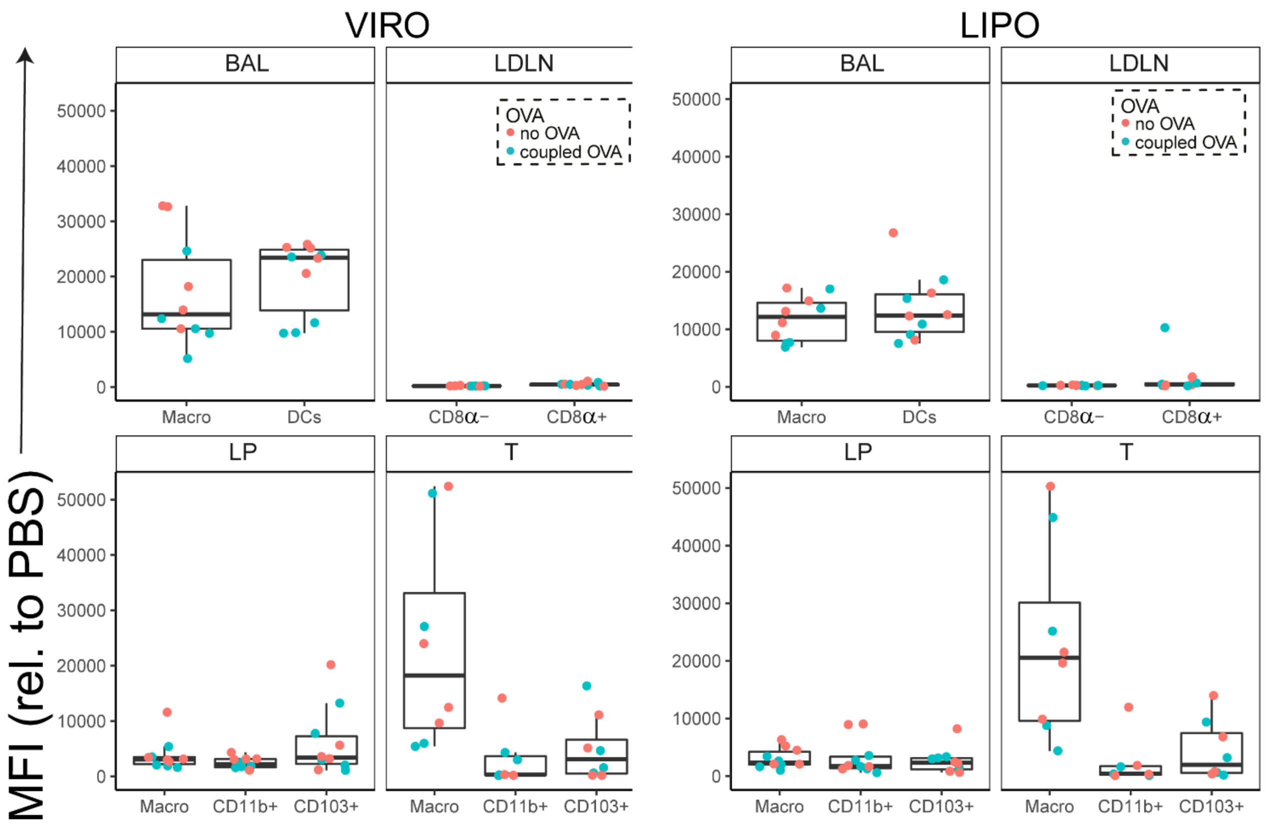

Supplementary Figure 2. Uptake of virosomes (VIRO) and liposomes (LIPO) by cells in the respiratory tract. Liposomes and virosomes without OVA ("no OVA") or with coupled OVA ("coupled OVA") were intra-nasally administered and cells from different lung compartments (BAL: broncho-alveolar lavage fluid, T: trachea, LP: lung parenchyma, LDLN: lung draining lymph nodes) were analyzed for uptake 24h later. Data represents MFI of uptake relative to PBS of five independent experiments. Statistical significance was determined by ANOVA followed by Tukey's HSD post hoc test to investigate individual paired comparisons.

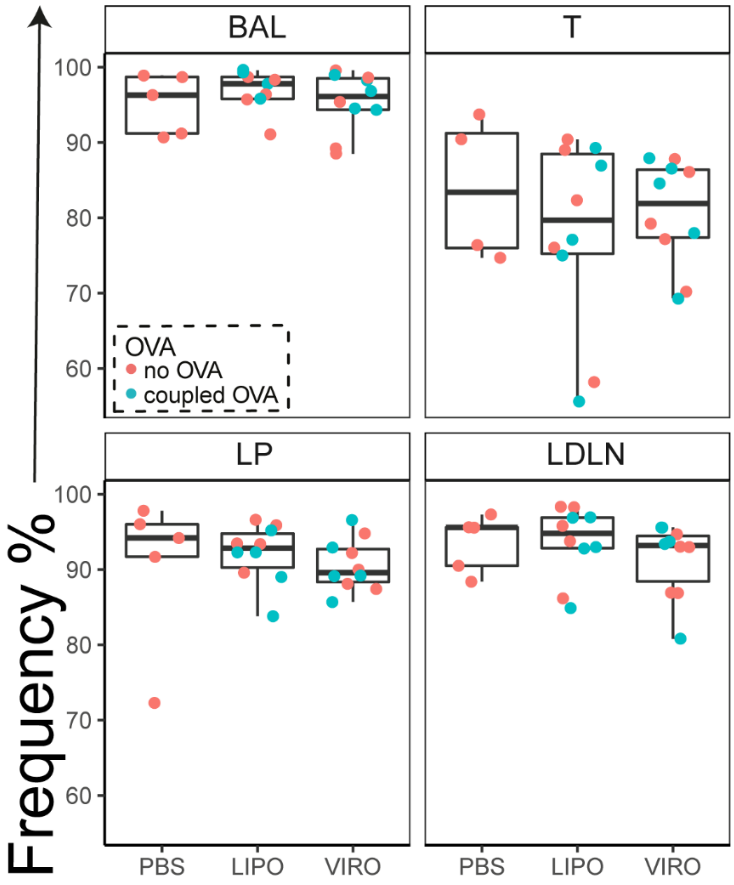

Supplementary Figure 3. Viability of cells after intra-nasal administration of liposomes (LIPO) and virosomes (VIRO) and digestion to single cell suspension. Liposomes and virosomes were intra-nasally applied to naïve BALB/c mice and lung compartments (BAL: broncho-alveolar lavage fluid, T: trachea, LP: lung parenchyma, LDLN: lung draining lymph nodes) harvested 24h later. Organs were digested and single cell suspension was prepared. Viability was controlled using a viability dye labeled with a fluorochrome and signal was measured by flow cytometry. Data represents five independent experiments. Statistical significance was determined by ANOVA followed by Tukey's HSD post hoc test to investigate individual paired comparisons.

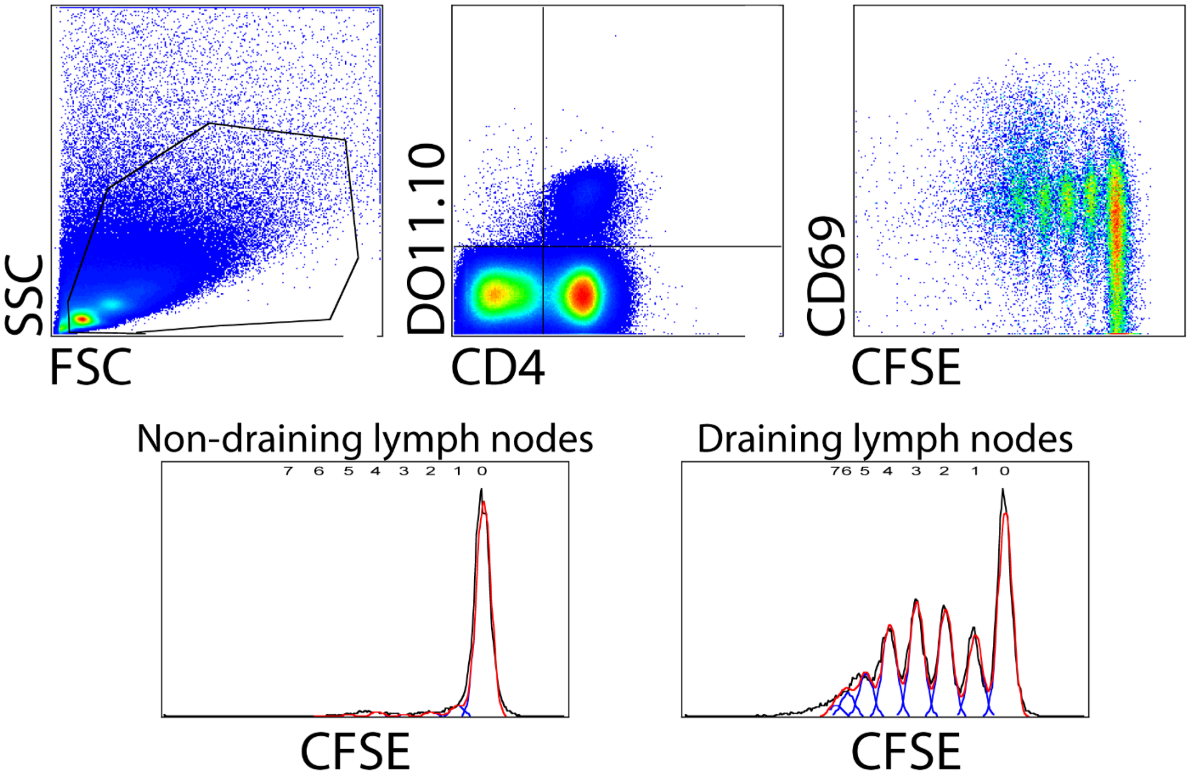

Supplementary Figure 4: FACS gating strategy for T cell proliferation. Cell gating included forward (FSC) and sideward (SSC) scatter for live cells followed by a $CD4^{+}$ $DO11.10^{+}$ gating. Double positive cells were analyzed for CD69 expression and CFSE profiles in NDLN (non-draining lymph nodes) and LDLN (lung-draining lymph nodes) to calculate the expansion index.

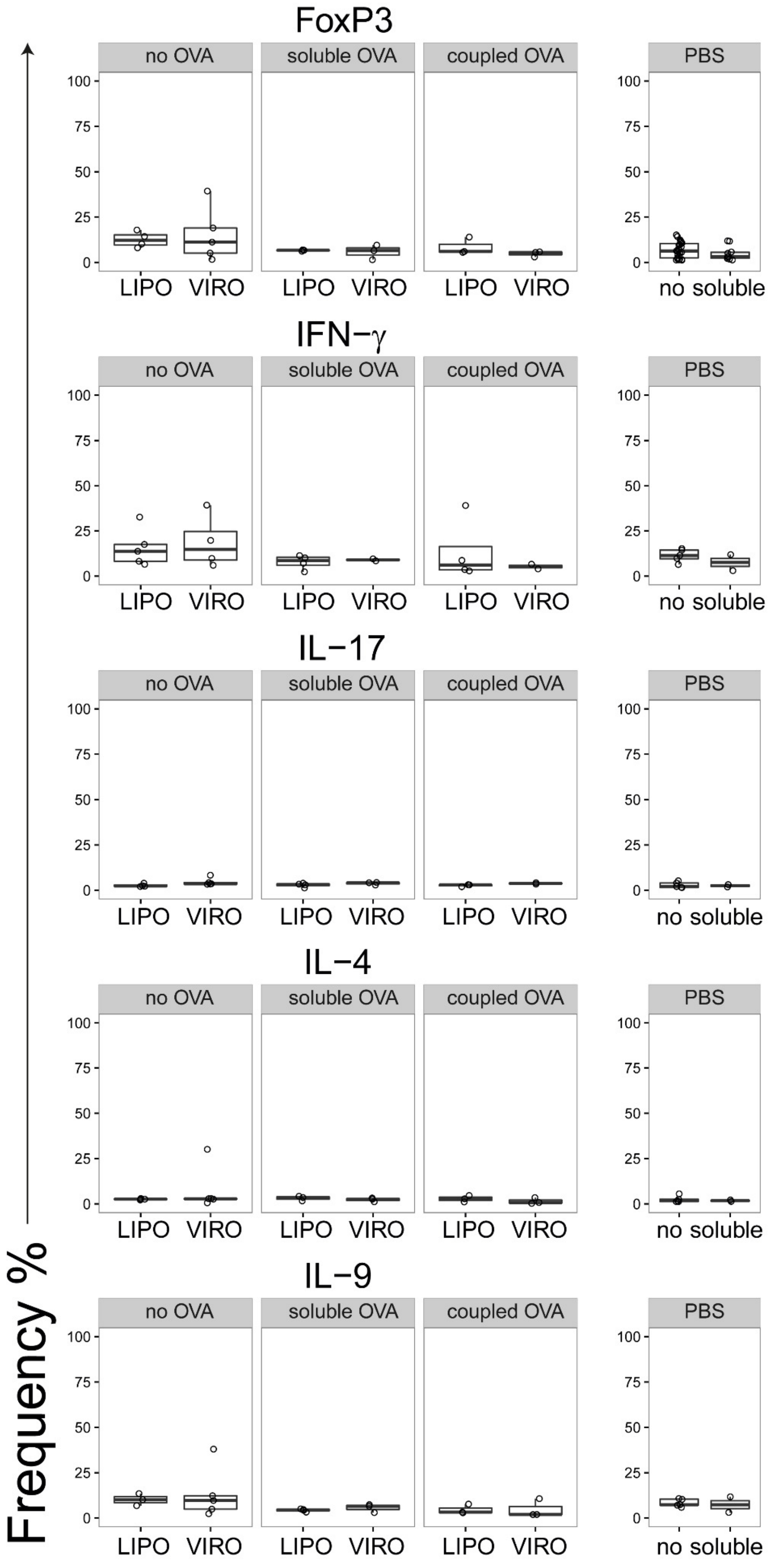
FoxP3
IFN−γ
IL−17
IL−4
IL−9
no OVA
soluble OVA
coupled OVA
PBS
LIPO
VIRO
no
soluble
Frequency %
0
25
50
75
100

Supplementary Figure 5: Measurement of antigen-specific CD4$^+$ T cell cytokine production by flow cytometry. CFSE-labeled CD4$^+$ T cells were injected intravenously in naïve BALB/c mice. After 48h, virosomes (VIRO), liposomes (LIPO) or PBS were given intra-nasally. 72h later LDLN were collected and stained for intracellular cytokines IFN-γ, IL-17, IL-4, IL-9 and transcription factor FoxP3. Figures show the frequency % of six independent experiments. Statistical significance was determined by ANOVA followed by Tukey's HSD post hoc test to investigate individual paired comparisons.

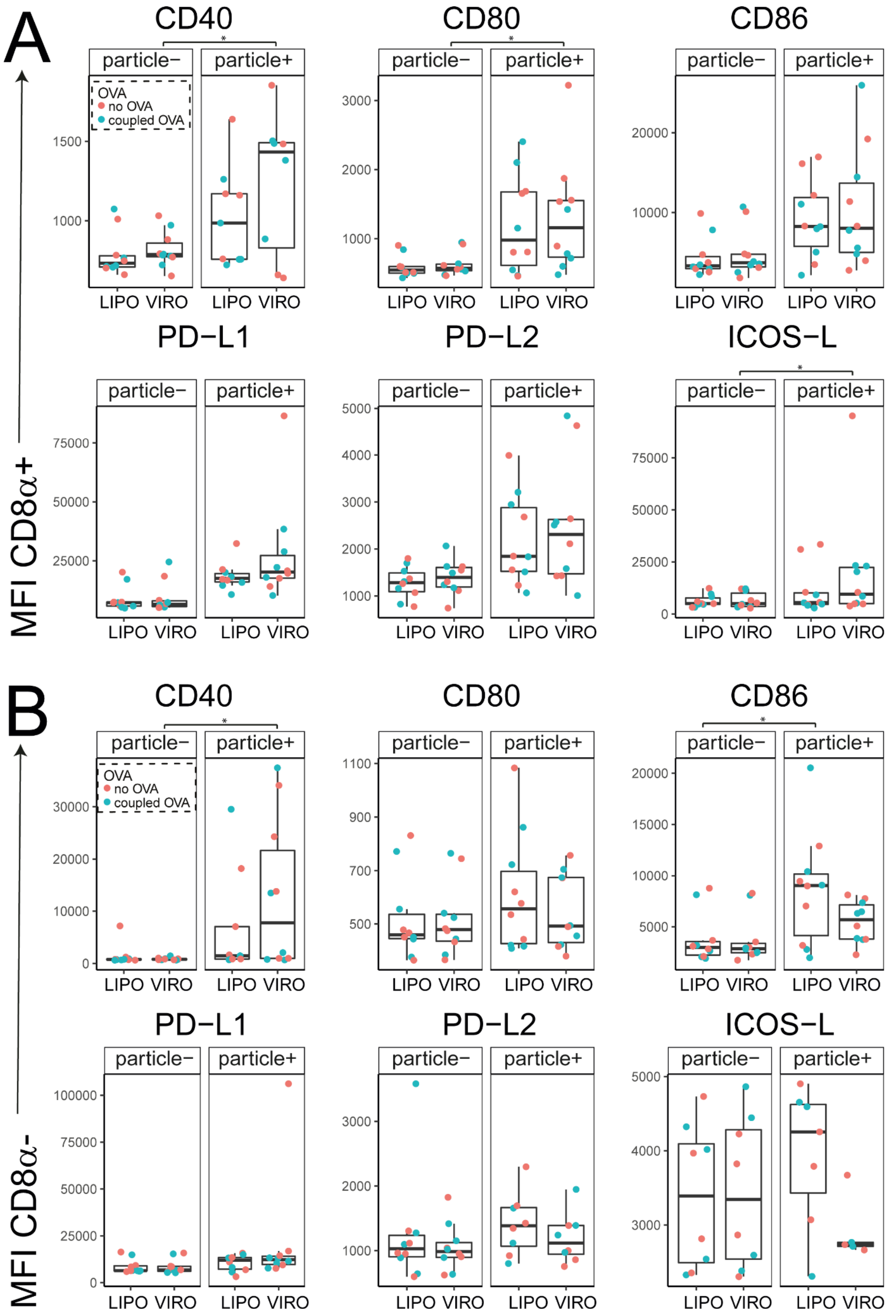
A
B
CD40
CD80
CD86
PD−L1
PD−L2
ICOS−L
particle−
particle+
OVA
no OVA
coupled OVA
LIPO VIRO
MFI CD8α+
MFI CD8α-

Supplementary Figure 6. Expression of surface markers in dendritic cells in lung-draining lymph nodes upon uptake of liposomes (LIPO) and virosomes (VIRO). Lung draining lymph nodes (LDLN) were harvested 24h after intra-nasal administration of empty liposomes or virosomes ("no OVA") or with liposomes and virosomes coupled to OVA ("coupled OVA") or PBS control (not shown). Particle negative (particle-) and particle positive (particle+) cell populations were analyzed for expression of surface markers CD40, CD80, CD86, PD-L1, PD-L2 and ICOS-L and measured by flow cytometry. Data shows MFI of expression of CD8α+ resident **(A)** and CD8α- migratory DCs **(B)** and represents five independent experiments. Statistical significance was determined by ANOVA followed by Tukey's HSD post hoc test to investigate individual paired comparisons. *p<0.05; **p<0.01; ***p<0.001.

.

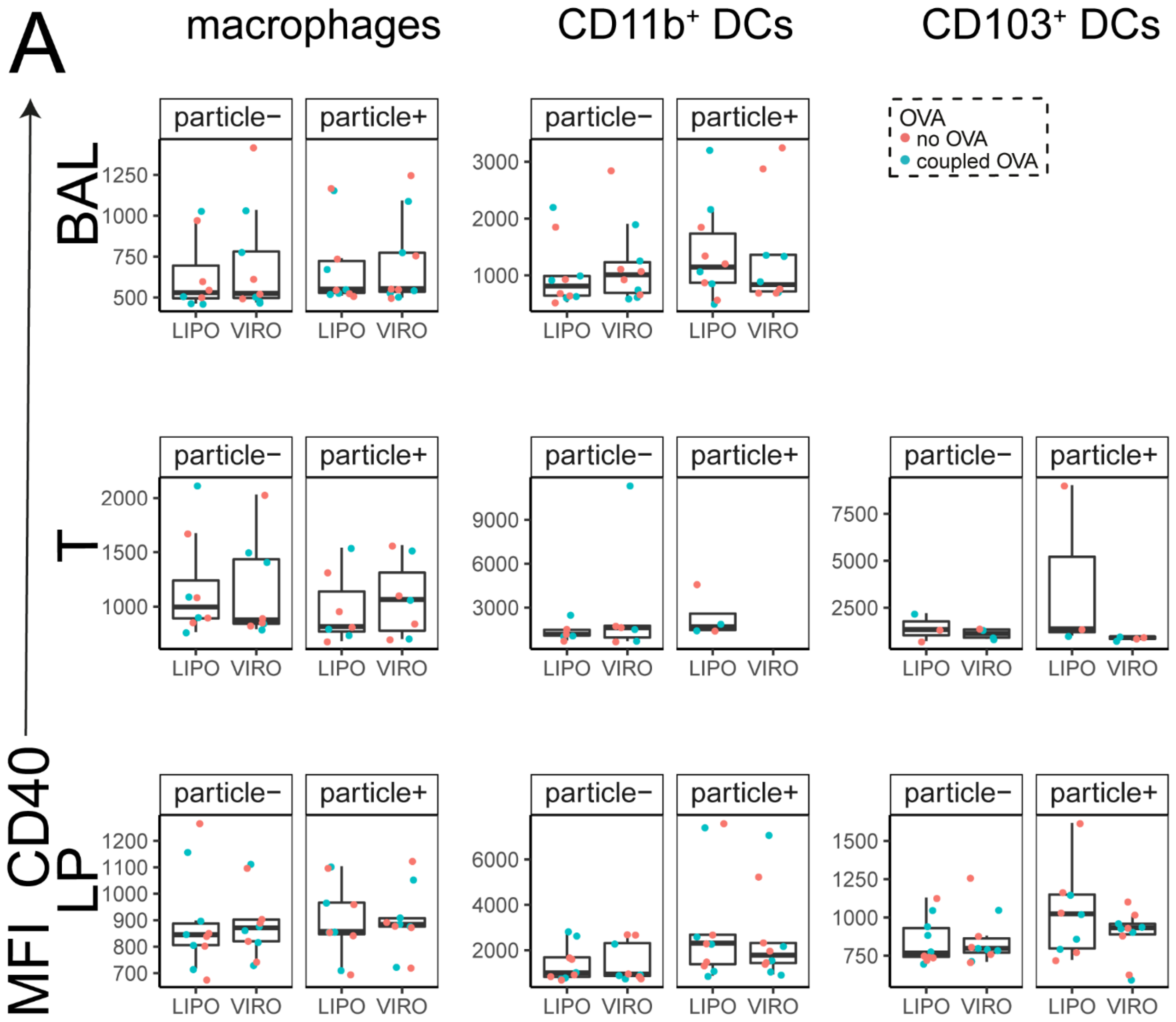
A
macrophages
CD11b+ DCs
CD103+ DCs
OVA
no OVA
coupled OVA
BAL
T
LP
MFI CD40
particle−
particle+
LIPO
VIRO

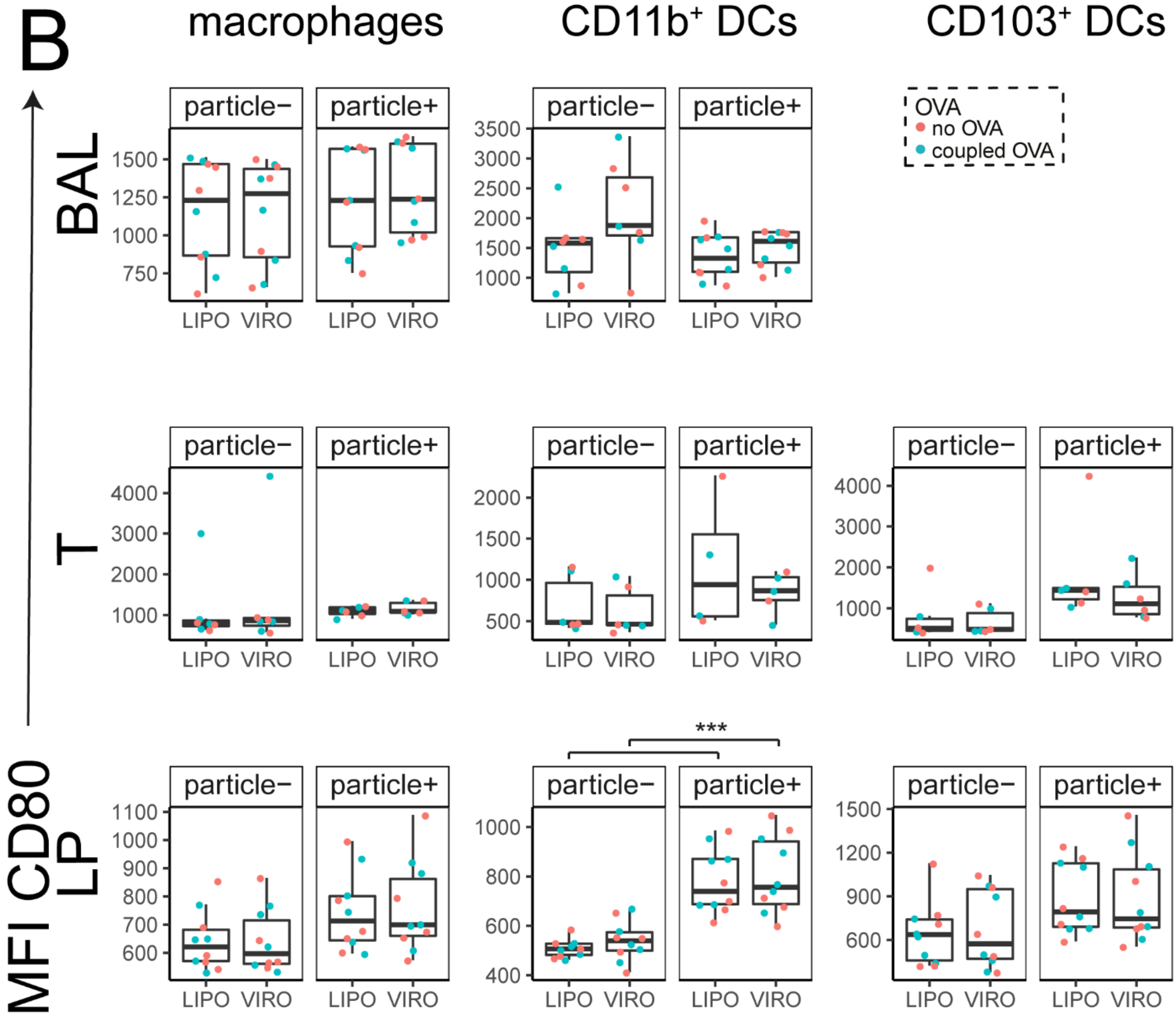

B
macrophages
CD11b+ DCs
CD103+ DCs
OVA
no OVA
coupled OVA
BAL
T
LP
MFI CD80
particle−
particle+
LIPO
VIRO
***

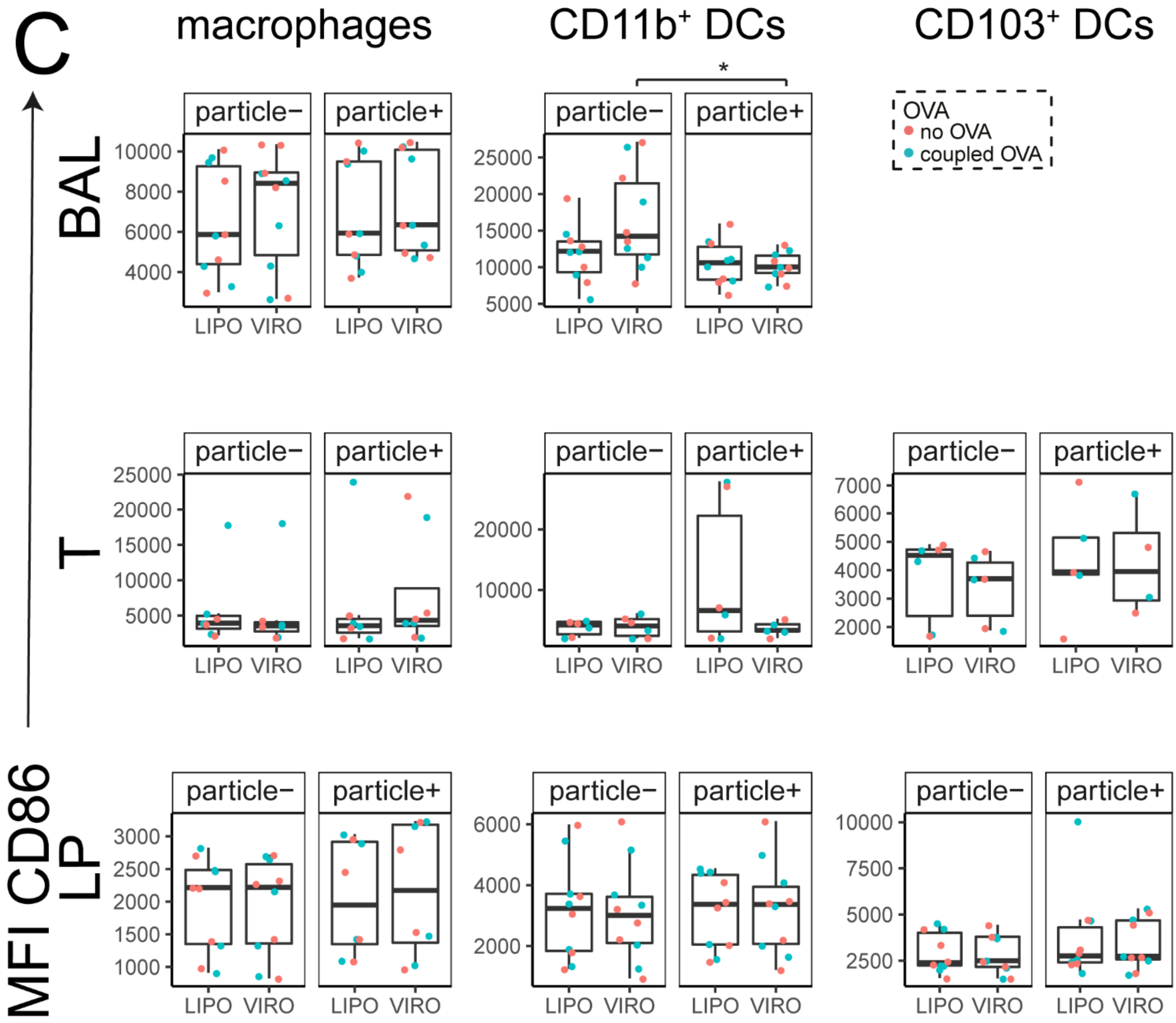
C
macrophages
CD11b+ DCs
CD103+ DCs
OVA
no OVA
coupled OVA
BAL
T
LP
MFI CD86
particle−
particle+
LIPO VIRO
*

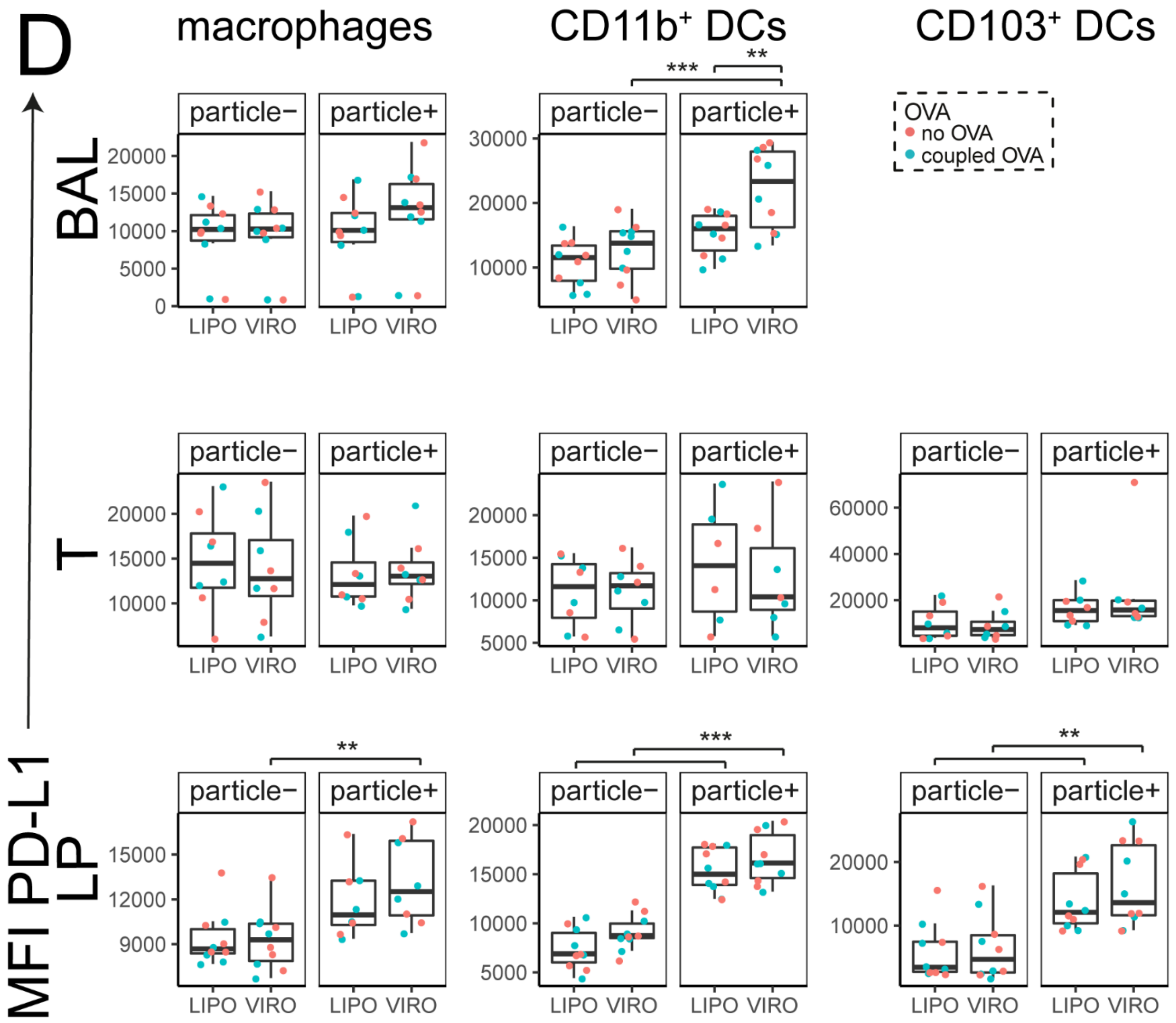
D
macrophages
CD11b+ DCs
CD103+ DCs
OVA
no OVA
coupled OVA
BAL
T
LP
MFI PD-L1
particle−
particle+
LIPO VIRO
***
**
20000
15000
10000
5000
0
30000
60000
40000
12000
9000

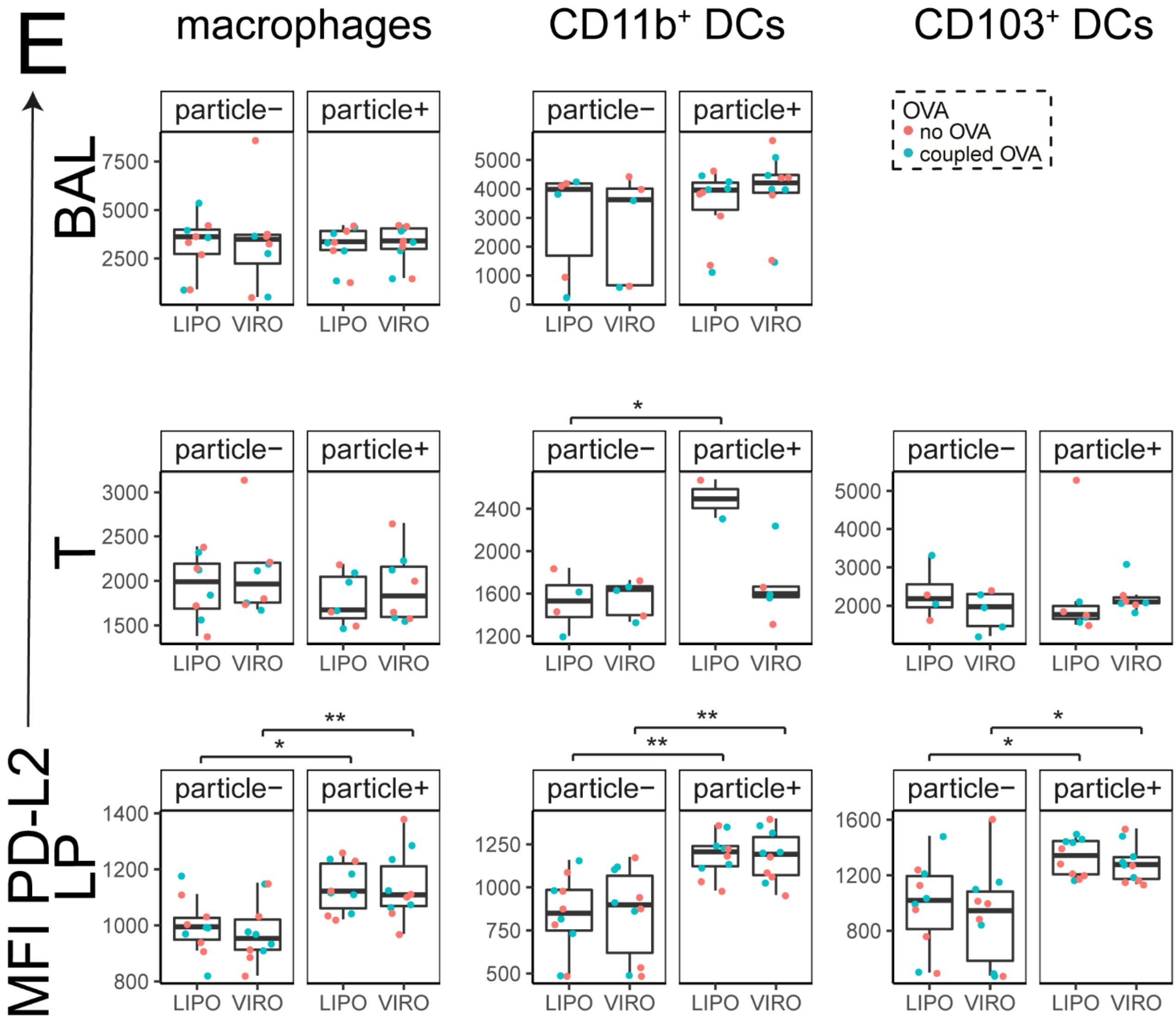
E
macrophages
CD11b+ DCs
CD103+ DCs
OVA
no OVA
coupled OVA
particle−
particle+
BAL
T
LP
MFI PD-L2
LIPO
VIRO
*
**

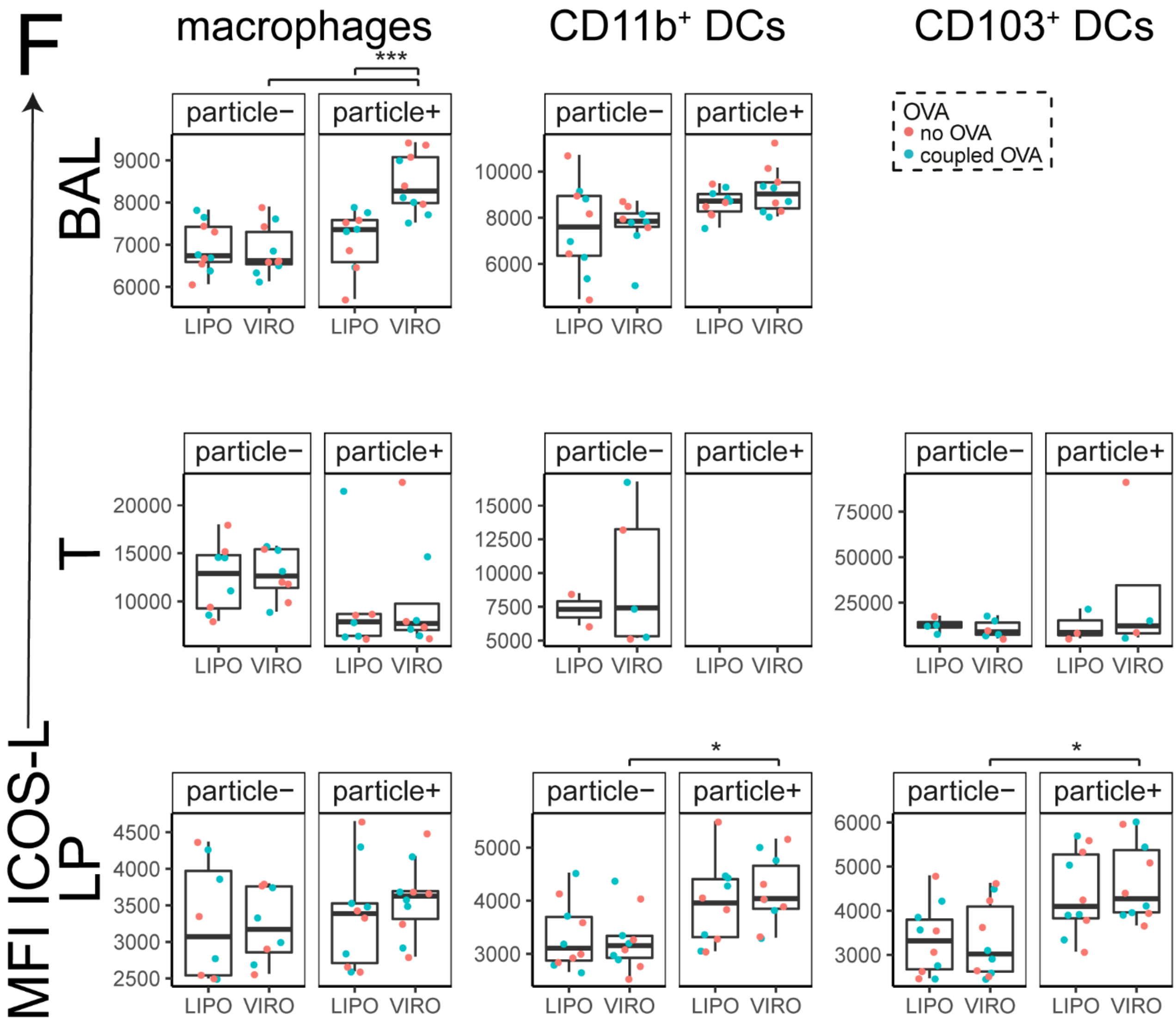

Supplementary Figure 7. Expression of surface markers in pulmonary antigen presenting cells upon uptake of liposomes (LIPO) and virosomes (VIRO). Lung compartments (BAL: broncho-alveolar lavage fluid, T: trachea, LP: lung parenchyma) were harvested 24h after intra-nasal administration of empty liposomes or virosomes ("no OVA") or with liposomes and virosomes coupled to OVA ("coupled OVA"). Particle negative (particle-) and particle positive (particle+) cell populations were analyzed for expression of surface markers CD40 **(A)**, CD80 **(B)**, CD86 **(C)**, PD-L1 **(D)**, PD-L2 **(E)** and ICOS-L **(F)** measured by flow cytometry and MFI is shown. Data represents five independent experiments. Statistical significance was determined by ANOVA followed by Tukey's HSD post hoc test to investigate individual paired comparisons. *p<0.05; **p<0.01; ***p<0.001.

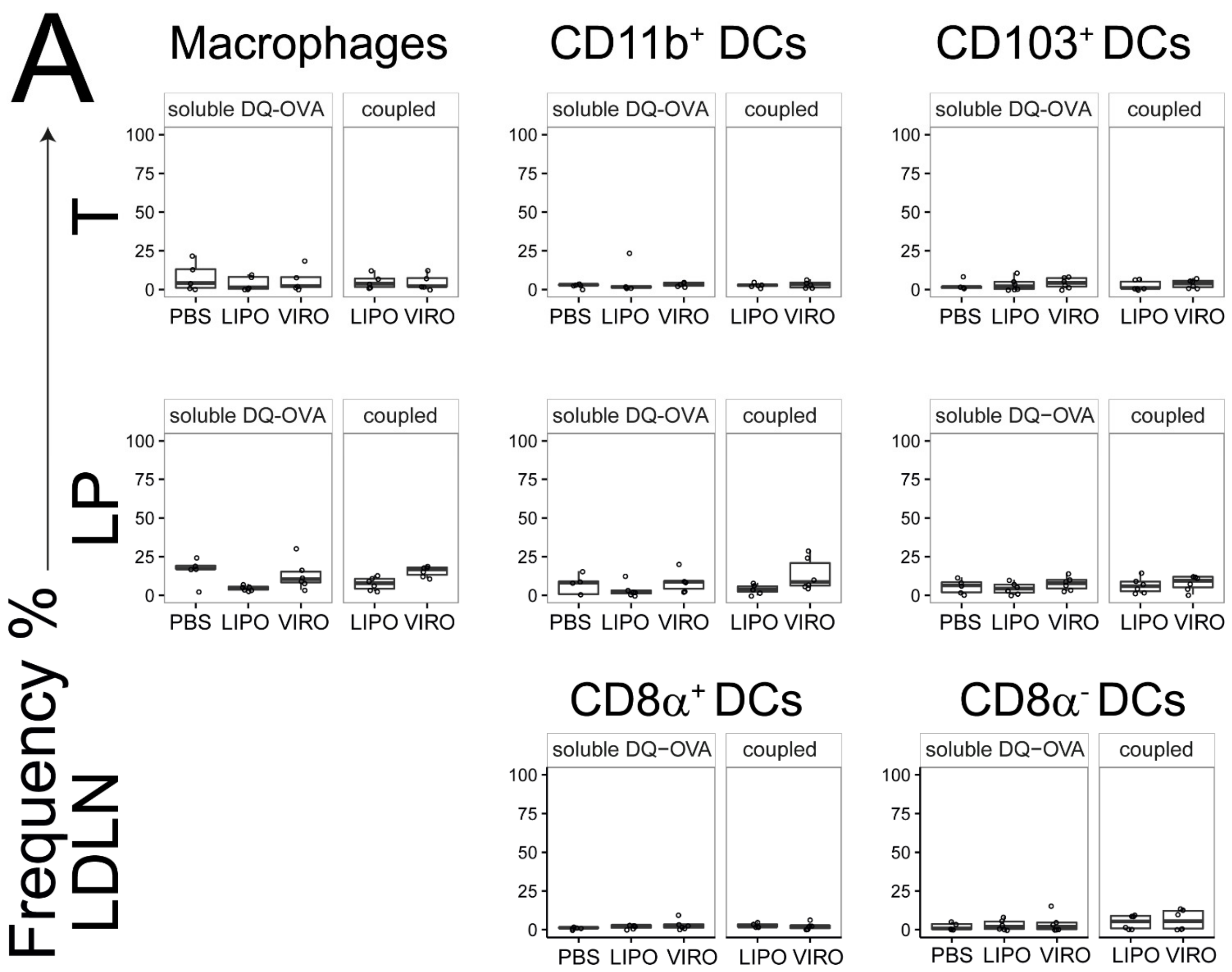
A
Macrophages
CD11b+ DCs
CD103+ DCs
soluble DQ-OVA
coupled
T
LP
Frequency %
LDLN
CD8α+ DCs
CD8α- DCs
PBS LIPO VIRO LIPO VIRO
100
75
50
25
0

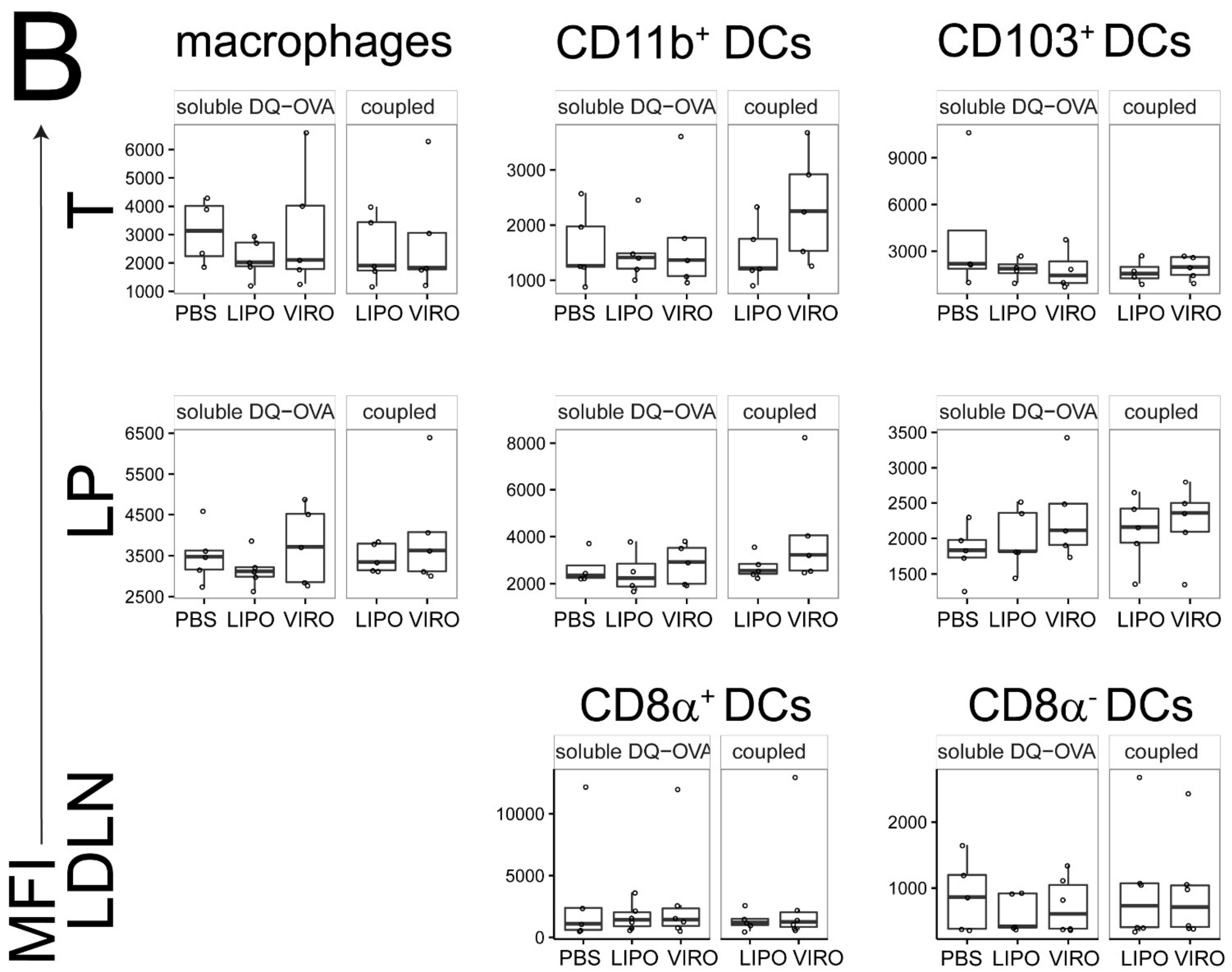

Supplementary Figure 8. Antigen degradation in pulmonary antigen presenting cells upon uptake of liposomes (LIPO) or virosomes (VIRO). Lung compartments (T, LP, LDLN) were harvested 24h after intra-nasal administration of empty liposomes, virosomes or PBS with soluble DQ-OVA ("soluble DQ-OVA") or with liposomes and virosomes coupled to DQ-OVA ("coupled"). Antigen degradation was analyzed by measuring released fluorochrome signal from self-quenching DQ-OVA by flow cytometry. Figures show the frequency **(A)** and MFI **(B)** of cells positive for degraded OVA relative to PBS. Data represents six independent experiments. Statistical significance was determined by ANOVA followed by Tukey's HSD post hoc test to investigate individual paired comparisons.

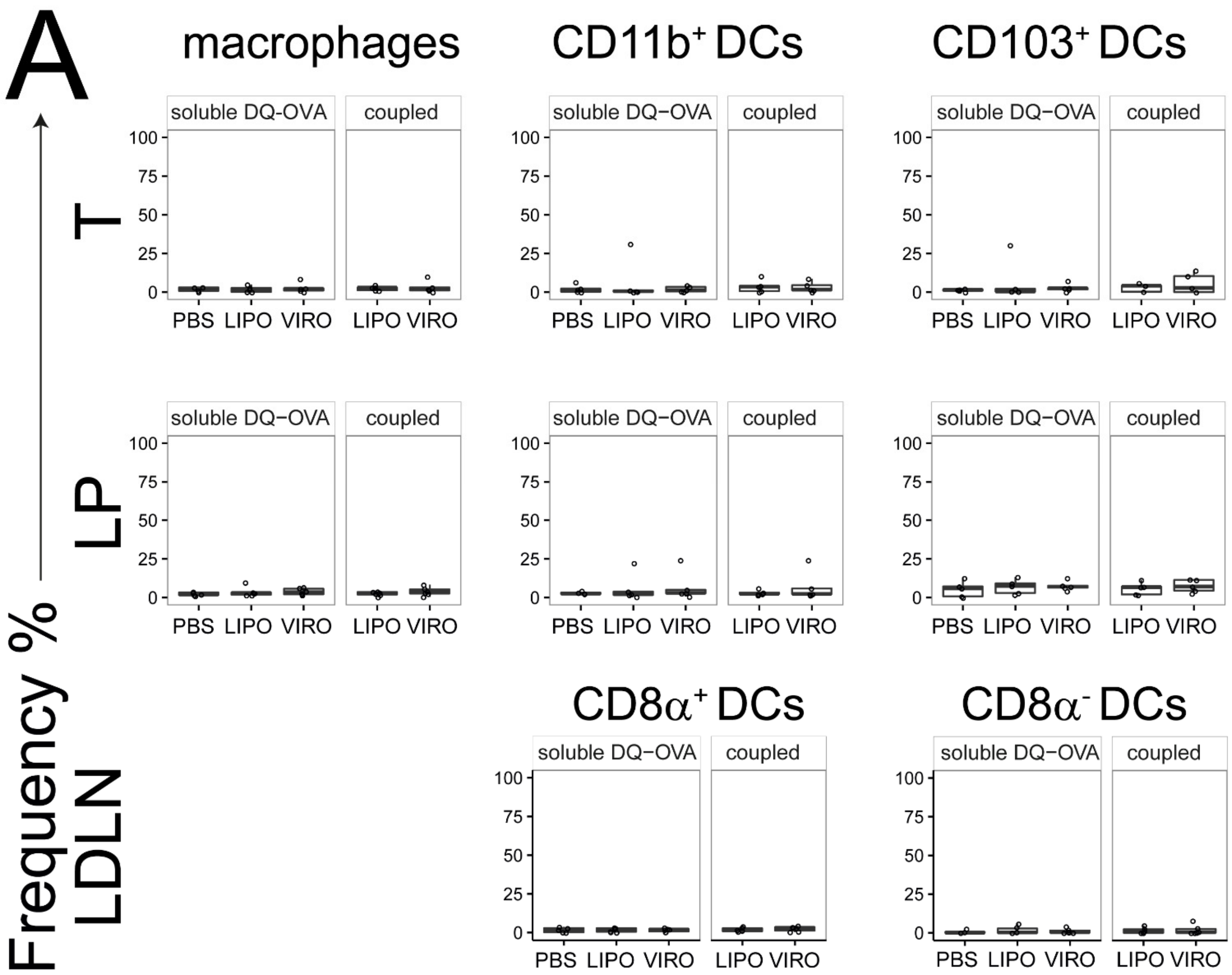
A
macrophages
CD11b+ DCs
CD103+ DCs
CD8α+ DCs
CD8α- DCs
soluble DQ-OVA
coupled
100
75
50
25
0
PBS LIPO VIRO LIPO VIRO
T
LP
LDLN
Frequency %

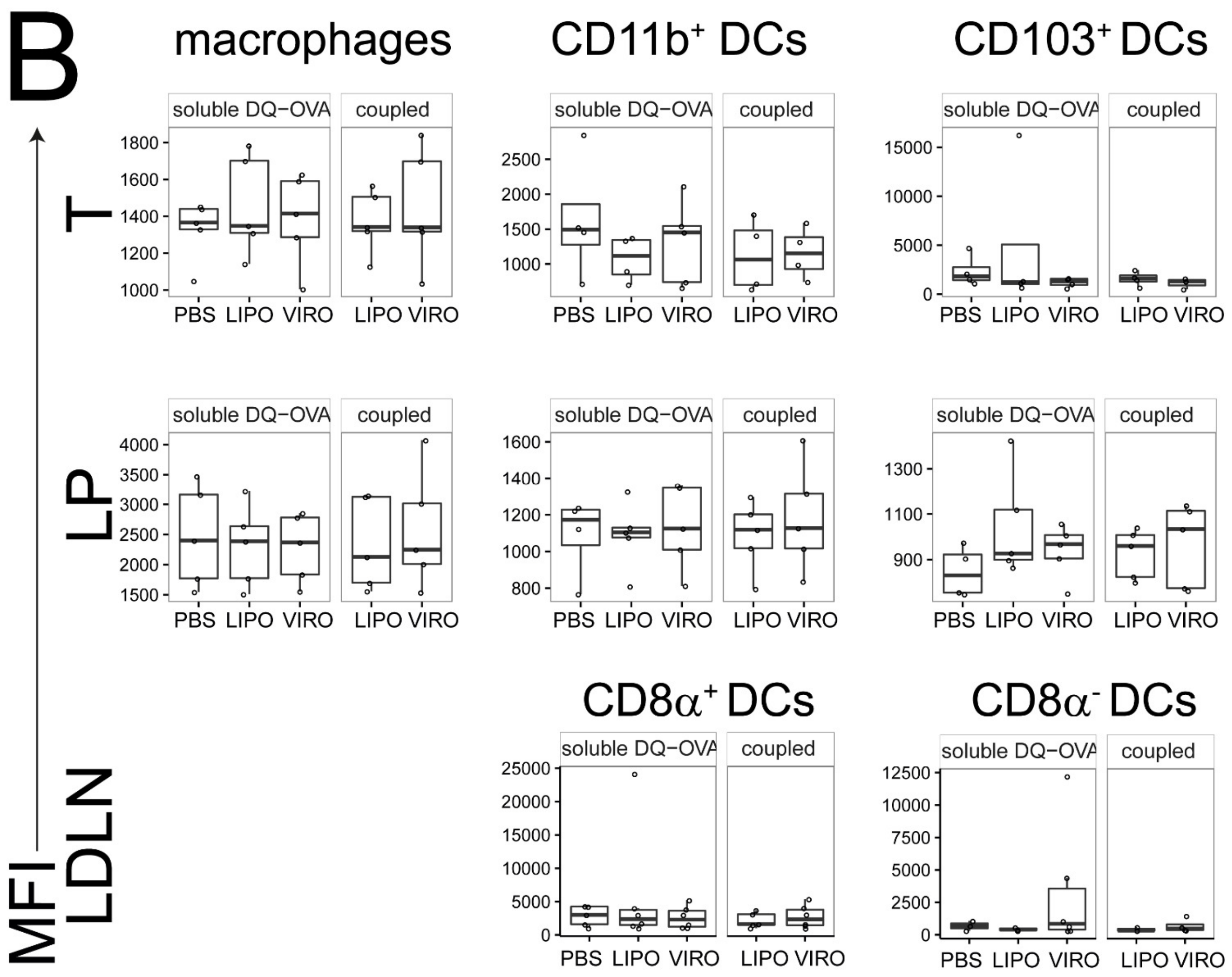

Supplementary Figure 9. Antigen accumulation in pulmonary antigen presenting cells upon uptake of liposomes (LIPO) or virosomes (VIRO). Lung compartments (T, LP, LDLN) were harvested 24h after intra-nasal administration of empty liposomes, virosomes or PBS with soluble DQ-OVA ("soluble DQ-OVA") or with liposomes and virosomes coupled to DQ-OVA ("coupled DQ-OVA"). Antigen accumulation was analyzed by measuring released fluorochrome signal from self-quenching DQ-OVA by flow cytometry. Figures show the frequency **(A)** and MFI **(B)** of cells positive for accumulated DQ-OVA relative to PBS control. Data represents six independent experiments. Statistical significance was determined by ANOVA followed by Tukey's HSD post hoc test to investigate individual paired comparisons.
